# Ammonia‐oxidising archaea living at low pH: Insights from comparative genomics

**DOI:** 10.1111/1462-2920.13971

**Published:** 2017-12-04

**Authors:** Craig W. Herbold, Laura E. Lehtovirta‐Morley, Man‐Young Jung, Nico Jehmlich, Bela Hausmann, Ping Han, Alexander Loy, Michael Pester, Luis A. Sayavedra‐Soto, Sung‐Keun Rhee, James I. Prosser, Graeme W. Nicol, Michael Wagner, Cécile Gubry‐Rangin

**Affiliations:** ^1^ Division of Microbial Ecology, Department of Microbiology and Ecosystem Science Research Network Chemistry meets Microbiology, University of Vienna Vienna Austria; ^2^ School of Biological Sciences University of East Anglia, Norwich Research Park Norwich NR4 7TJ UK; ^3^ School of Biological Sciences University of Aberdeen, Cruickshank Building, St Machar Drive Aberdeen AB24 3UU UK; ^4^ Department of Microbiology Chungbuk National University, 1 Chungdae‐ro, Seowon‐Gu Cheongju 362‐763 South Korea; ^5^ Department of Molecular Systems Biology Helmholtz‐Centre for Environmental Research‐UFZ Leipzig 04318 Germany; ^6^ Department of Microorganisms Leibniz Institute DSMZ ‐ German Collection of Microorganisms and Cell Cultures Inhoffenstr. 7B Braunschweig 38124 Germany; ^7^ Department of Botany and Plant Pathology Oregon State University, 2082 Cordley Hall Corvallis OR 97331‐2902 USA; ^8^ Laboratoire Ampère, École Centrale de Lyon, L'Université de Lyon, 36 avenue Guy de Collongue 69134 Ecully CEDEX France

## Abstract

Obligate acidophilic members of the thaumarchaeotal genus *Candidatus* Nitrosotalea play an important role in nitrification in acidic soils, but their evolutionary and physiological adaptations to acidic environments are still poorly understood, with only a single member of this genus (*Ca*. N. devanaterra) having its genome sequenced. In this study, we sequenced the genomes of two additional cultured *Ca*. Nitrosotalea strains, extracted an almost complete *Ca*. Nitrosotalea metagenome‐assembled genome from an acidic fen, and performed comparative genomics of the four *Ca*. Nitrosotalea genomes with 19 other archaeal ammonia oxidiser genomes. Average nucleotide and amino acid identities revealed that the four *Ca*. Nitrosotalea strains represent separate species within the genus. The four *Ca*. Nitrosotalea genomes contained a core set of 103 orthologous gene families absent from all other ammonia‐oxidizing archaea and, for most of these gene families, expression could be demonstrated in laboratory culture or the environment via proteomic or metatranscriptomic analyses respectively. Phylogenetic analyses indicated that four of these core gene families were acquired by the *Ca*. Nitrosotalea common ancestor via horizontal gene transfer from acidophilic representatives of Euryarchaeota. We hypothesize that gene exchange with these acidophiles contributed to the competitive success of the *Ca*. Nitrosotalea lineage in acidic environments.

## Introduction

Nitrification, the oxidation of ammonia to nitrate via nitrite, is a central process within the terrestrial nitrogen cycle, determining the form of inorganic nitrogen available to plants, decreasing nitrogen fertilizer utilization efficiency and contributing to atmospheric and groundwater pollution by nitrous oxide and nitrate respectively (Robertson and Vitousek, 2009). Nitrification in soil is generally limited by the initial oxidation of ammonia to nitrite, in which archaeal ammonia oxidisers play a significant role (e.g., Lu *et al*., 2015; Hink *et al*., 2017). Net rates of nitrification do not show a strong correlation with soil pH and some of the highest rates are found in acidic soils (pH < 5) (Booth *et al*., 2005), which comprise approximately 30% of all soils (von Uexküll and Mutert, 1995). Surveys of 16S rRNA and ammonia monooxygenase subunit A (*amoA*) genes demonstrate that ammonia oxidising archaea (AOA) are distributed globally in soils, with pH being an important driver of both community composition and adaptation (Gubry‐Rangin *et al*., 2011; Gubry‐Rangin *et al*., 2015; Vico Oton *et al*., 2016).

Genome‐wide prediction of the functional adaptation of ammonia oxidising Thaumarchaeota to low pH has thus far been limited to the genome of the soil isolate *Candidatus* Nitrosotalea devanaterra (Lehtovirta‐Morley *et al*., 2016). This genomic analysis identified potential mechanisms for substrate acquisition and pH homeostasis in acidic environments that present potential constraints for ammonia oxidisers. The concentration of NH_3_, the most likely substrate for bacterial and archaeal ammonia monooxygenases, is significantly reduced at pH values below 7, as the p*K*_a_ for the ${\text{NH}}_{4}^{+}$ ⇆ NH_3_ equilibrium is 9.25. Decreasing pH also moves the ${\text{NO}}_{2}^{-}$ ⇆ HNO_2_ equilibrium toward inhibitory nitrous acid, which is highly reactive, with breakdown products that can cause extensive cellular damage. In addition, growth at low pH requires mechanisms for pH homeostasis, to maintain the transmembrane proton gradient required for ATP production and normal function of cellular processes. Genome and cell membrane analyses indicated that such mechanisms might exist in *Ca*. N. devanaterra, including cation uptake, cytoplasmic buffering and a cell membrane composition distinct from that of neutrophilic AOA (Lehtovirta‐Morley *et al*., 2016). In addition, Amt‐type ${\text{NH}}_{4}^{+}$ transporters are predicted to be encoded by all sequenced AOA genomes (including *Ca*. N. devanaterra) and are distinct from Rh‐type NH_3_ transporters found in some ammonia oxidising bacteria (AOB) (Offre *et al*., 2014; Lehtovirta‐Morley *et al*., 2016). Ammonia or ammonium is required for both energy generation and nitrogen assimilation by ammonia oxidisers, and the preference of *Ca*. N. devanaterra (and other AOA) for transporting ${\text{NH}}_{4}^{+}$ may contribute to its ability to grow in acidic environments containing limiting concentrations of NH_3_. In this context, it is interesting to note that the recently isolated acid‐adapted (growth in the range of pH 5–7.5) gammaproteobacterial AOB *Ca*. Nitrosoglobus terrae does not encode known transporters for ${\text{NH}}_{4}^{+}$ or NH_3_ and might, thus, rely on passive diffusion of ammonia through its membrane for assimilation (Hayatsu *et al*., 2017).

While the genome of *Ca*. N. devanaterra has allowed the generation of hypotheses regarding mechanisms facilitating its unique physiology, the absence of further acidophilic archaeal ammonia oxidiser genomes made it difficult to confirm these findings. The aim of this study was to gain a greater understanding of the function and origin of the genes potentially involved in acidophilic adaptation in the *Ca*. genus Nitrosotalea through comparative genomics by including three newly determined genomes from this genus, and to learn whether these genes are expressed under natural and/or cultivation conditions. Specifically, this study aimed to reveal (1) the (compositional) similarity of genomes within *Ca*. Nitrosotalea and with those of other AOA; (2) the size and predicted function of the *Ca*. Nitrosotalea core genome compared to that of other AOA genera; (3) whether the *Ca*. Nitrosotalea core genome is expressed; and (4) the evolutionary origin(s) of gene families that comprise the *Ca*. Nitrosotalea core genome.

## Results

### Expanded genomic representation of *Candidatus* Nitrosotalea

In this study, the genomes from two cultured strains of *Ca*. Nitrosotalea, strain Nd2 (Lehtovirta‐Morley *et al*., 2014) and strain CS (Jung *et al*., 2014), were sequenced. In addition, a *Ca*. Nitrosotalea metagenome‐assembled genome (strain SbT1) was recovered from an acidic fen, anaerobic, stable isotope probing experiment (Pester *et al*., 2012; Hausmann *et al*., 2016) (Supporting Information 1 and Fig. S1).

### Molecular evidence for four *Candidatus* Nitrosotalea species

**Figure 1:**
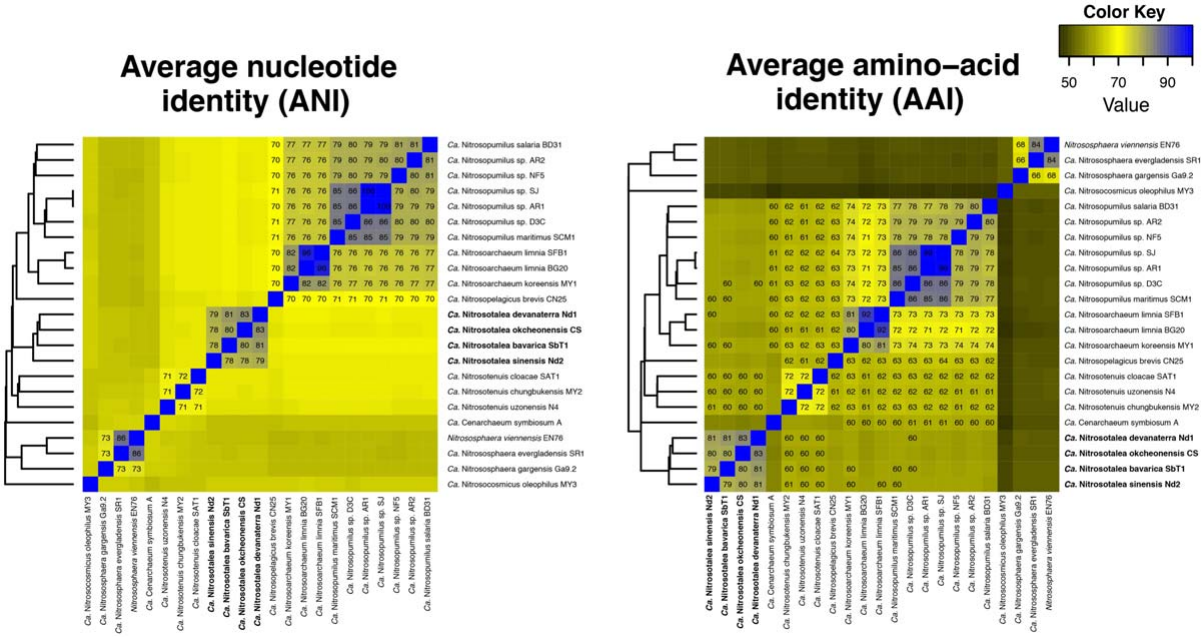
Heat maps showing pairwise ANI and AAI values inferred from the four *Ca*. Nitrosotalea genomes (bold) and other available Thaumarchaeota genomes. Dendrograms were calculated by hierarchical clustering (hclust, method = complete linkage) in *R* with distances calculated as (100% – %identity, that is, 100% ANI = 0 distance). For ANI and AAI, values above 70% and 60%, respectively, are indicated on the heat map. [Colour figure can be viewed at wileyonlinelibrary.com]

The 16S ribosomal RNA gene sequences of the four *Ca*. Nitrosotalea strains exceed 99% nucleotide identity to one another and are thus not useful for elucidating precise taxonomic relationships among these strains (Yarza *et al*., 2014). Genomes of the four strains were therefore compared by determining average amino acid and nucleotide identities (AAI and ANI; Fig. 1). AAI between the four genomes were 79%–83% with >80% of genes aligned, suggesting they represent different species of the same genus (Luo *et al*., 2014). Likewise, ANI values between the four genomes were 78%–83%, far below the proposed species delineation boundaries of 95%–97% (Goris *et al*., 2007; Varghese *et al*., 2015). Therefore, we propose that each of the four analysed strains represents a separate species within the *Ca*. Nitrosotalea genus.

### Phylogenomic relationship of *Candidatus* Nitrosotalea with other AOA

**Figure 2:**
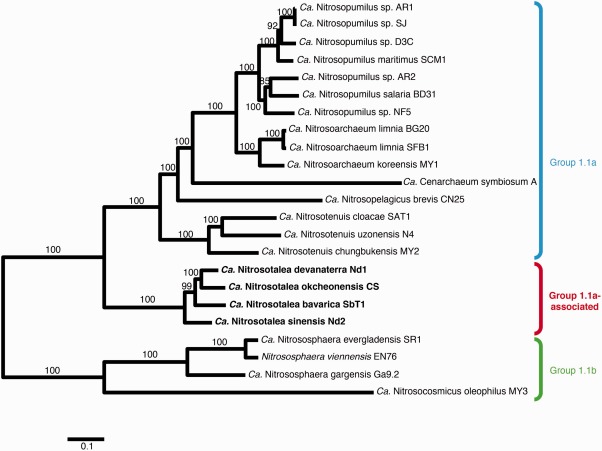
Phylogenetic relationship of *Ca*. Nitrosotalea genomes (bold) with other sequenced AOA based on a RaxML phylogenetic analysis of 198 concatenated single‐copy universal arCOG markers. Bootstrap values for internal branches are shown. [Colour figure can be viewed at wileyonlinelibrary.com]

In single gene trees based on 16S rRNA (Supporting Information Fig. S2a) and *amoA* (Supporting Information Fig. S2b) genes, the four *Ca*. Nitrosotalea species formed a monophyletic sister group to group 1.1a Thaumarchaeota (*Ca*. Nitrosopumilus, *Ca*. Nitrosoarchaeum and *Ca*. Nitrosotenuis), consistent with previous placements of this genus (Lehtovirta‐Morley *et al*., 2011; Pester *et al*., 2012; Vico Oton *et al*., 2016). For more refined analyses, two concatenated sets of marker genes (a ‘universal’ marker set consisting of 34 genes (Parks *et al*., 2015) and a set of 198 single‐copy genes that are phylogenetically congruent among all AOA, see Supporting Information Table SI.2.1) were also used to infer the phylogenetic relationship of the four *Ca*. Nitrosotalea species with other fully sequenced AOA genomes. Again, *Ca*. Nitrosotalea was consistently recovered as a monophyletic sister group to group 1.1a, distinct from 1.1b taxa (*Nitrososphaera*, *Ca*. Nitrosocosmicus; Bayesian *P* > 0.999 and bootstrap support = 1; Fig. 2 and Supporting Information Fig. S2c). The only major disagreement between these two trees is in the branching order of lineages represented by *Ca*. Cenarchaeum symbiosum and *Ca*. Nitrosopelagicus brevis (Fig. 2 and Supporting Information Fig. S2c). As both lineages are currently represented by a single member, addition of sister taxa to these two relatively long‐branch taxa may help resolve the disagreement.

### Thaumarchaeota and *Candidatus* Nitrosotalea core genomes

The quality of the sampled genomes and the phylogenetic breadth of groups used for comparison strongly influence core genome analyses. Genome completeness, open reading frame predictions and AAI‐based grouping of genomes were therefore considered *a priori* in the description of core genomes. Genome completeness, assessed by two methods, was high (>92%) for all 23 AOA genomes (Table 1) but many contain pathway gaps, likely to be artefacts of different gene‐calling approaches. Gene‐calling was therefore repeated for all AOA genomes using Prodigal (Hyatt *et al*., 2010) and resulted in a much larger core genome shared by all 23 AOA (640 and 743 using gene calls from GenBank and *de novo* gene calls made by Prodigal respectively). Prodigal gene calls were thus used in subsequent analyses. AAI between genomes varied extensively within and between groups (Fig. 1). The range of proposed genus‐level cut‐offs for AAI (60%–80%; Luo *et al*., 2014) is inconsistent with the currently used AOA taxonomy. For instance, at 60% AAI *Ca*. Nitrosotenuis, *Ca*. Nitrosopumilus, *Ca*. Nitrosopelagicus and *Ca*. Nitrosoarchaeum would form a single genus, at 70% AAI, *Nitrososphaera* would be split into two genera, while *Ca*. Nitrosopumilus and *Ca*. Nitrosoarchaeum would form a single genus. At 80% *Nitrososphaera, Ca*. Nitrosopumilus and *Ca*. Nitrosotenuis would split into multiple genera. This information was considered when comparing genus‐specific core gene sets (see below).

**Table 1:** AOA used for the comparative genome analysis.

		Completeness (%)	
Organism	Source/accession number	arCOG	CheckM ^a^
*Ca*. Nitrosopumilus maritimus SCM1^b^	CP000866.1	100.00	100.0 (0.97)
*Ca*. Nitrosopumilus sp. SJ	NZ_AJVI00000000.1	97.50	96.12 (0)
*Ca*. Nitrosopumilus sp. AR1	CP003842.1	96.67	94.66 (0)
*Ca*. Nitrosopumilus sp. AR2	CP003843.1	97.50	97.09 (0)
*Ca*. Nitrosopumilus salaria BD31	NZ_AEXL00000000.2	92.50	92.39 (1.94)
*Ca*. Nitrosopumilus sp. D3C	CP010868.1	100.00	100.0 (0.97)
*Ca*. Nitrosopumilus sp. NF5	CP011070.1	100.00	100.0 (0)
*Ca*. Nitrosoarchaeum koreensis MY1	AFPU01000001.1	100.00	100.0 (0)
*Ca*. Nitrosoarchaeum limnia BG20	NZ_AHJG00000000.1	100.00	99.03 (5.83)
*Ca*. Nitrosoarchaeum limnia SFB1	CM001158.1	99.17	98.06 (0)
*Ca*. Nitrosopelagicus brevis CN25	NZ_CP007026.1	100.00	99.51 (0)
*Ca*. Cenarchaeum symbiosum A	DP000238.1	98.33	99.03 (0)
*Ca*. Nitrosotenuis uzonensis N4	NZ_CBTY000000000.1	100.00	100.0
*Ca*. Nitrosotenuis chungbukensis MY2	NZ_AVSQ00000000.1	98.33	99.03 (0.97)
*Ca*. Nitrosotenuis cloacae SAT1	CP011097.2	99.17	100.0
*Ca*. Nitrosotalea okcheonensis CS	ERS1465380	99.17	99.51 (0)
*Ca*. Nitrosotalea sinensis Nd2	ERS1465381	100.00	99.51 (0.97)
*Ca*. Nitrosotalea devanaterra Nd1^b^	ERS884509	100.00	98.54 (0)
*Ca*. Nitrosotalea bavarica SbT1	ERS1572876	98.33	96.60 (0.97)
*Ca*. Nitrosocosmicus oleophilus MY3	CP012850.1	99.17	98.06 (0.97)
*Nitrososphaera viennensis* EN76^b^	CP007536.1	100.00	100.0 (0.97)
*Ca*. Nitrososphaera evergladensis SR1^b^	CP007174.1	100.00	100.0 (2.91)
*Ca*. Nitrososphaera gargensis Ga9.2^b^	CP002408.1	100.00	100.0 (2.91)

a. In addition to the genomic completeness, CheckM software predicts the level of genomic contamination (in brackets) as a proportion of multiple copies, in the genome of interest, of known conserved single‐copy genes in closely related genomes.

b. Closed genomes.

Predicted genes from the 23 AOA genomes (including the four *Ca*. Nitrosotalea genomes) were clustered into 11,655 orthologous gene families using OrthoMCL (Li *et al*., 2003), of which 4,868 gene families were unique to single taxa and 743 were found in all genomes, forming a thaumarchaeotal core genome (Supporting Information 1 and Table SI.2.1). As expected, this number is lower than the 860 core genome gene families of Thaumarchaeota recently reported by Kerou *et al*. (2016) reflecting our inclusion of more genomes and use of different cut‐off values and algorithms. Of the 743 gene families of the thaumarchaeotal core genome determined in this study, 697 were also retrieved by Kerou *et al*. (Supporting Information Table SI.2.2).

*Ca*. Nitrosotalea genes were present in 2,902 gene families, almost half of which (1,363) were common to all four *Ca*. Nitrosotalea genomes. The *Ca*. Nitrosotalea‐specific core genome comprised 103 orthologous gene families restricted within the Thaumarchaeota to *Ca*. Nitrosotalea (Supporting Information 1 and Table SI.2.3). This was lower than the respective *Nitrososphaera‐*specific core genome, whether including *Ca*. N. gargensis (331 gene families) or excluding it (333 gene families) due to low shared AAI with other members of this genus. Their larger core genome likely reflects the greater genome size of genus *Nitrososphaera* members. The *Ca*. Nitrosotalea‐specific core is, however, much larger than that of group 1.1a AOA (Supporting Information Table SI.2.1), contrasting with only 10, 23 and 40 gene families for the *Ca*. Nitrosopumilus‐specific core, the *Ca*. Nitrosoarchaeum‐specific core and the combined *Ca*. Nitrosopumilus*/Ca*. Nitrosoarchaeum‐specific core respectively. To account for sampling bias in core‐genome definitions, AAI was used to select combinations of four dissimilar *Ca*. Nitrosopumilus*/Ca*. Nitrosoarchaeum genomes to mimic the diversity of *Ca*. Nitrosotalea. This resulted in a maximum of 28 *Ca*. Nitrosopumilus*/Ca*. Nitrosoarchaeum‐specific core gene families.

### Origin of gene families in the *Candidatus* Nitrosotalea‐specific core genome

The potential evolutionary origin of the 103 orthologous gene families (comprising 420 genes) identified as the ‘*Ca*. Nitrosotalea*‐*specific core’ was examined based on phylogenetic tree topology‐based inference. Of these 103 gene families, seven shared homology with gene families present in non‐AOA microbes but not in other AOA; 38 showed little or no homology (<30% amino acid identity) to any other gene families in other AOA or to any other sequences in the NCBI GenBank nr protein database; 12 returned only one to three low scoring (30%–45% amino acid identity) hits in Blast‐based searches, preventing further phylogenetic analysis; and 46 shared homology with other gene families in Thaumarchaeota (>30% amino acid similarity between members of each gene family). These 46 gene families were nevertheless inferred to be *Ca*. Nitrosotalea‐specific in the OrthoMCL‐based approach because the pairwise similarity and connectivity between the members of each gene family was insufficient to assign them confidently to common orthologous groups with other AOA (Table 2). In manually examined phylogenetic trees (not shown), all but one of this subset of 46 *Ca*. Nitrosotalea*‐*specific core gene families branch with other AOA. Phylogenetic reconstructions for eight gene families (one with homology in other AOAs and seven absent from other AOAs), suggested HGT events involving a common ancestor of the *Ca*. Nitrosotalea (Table 2 and Supporting Information Figs S3–S10). Five of these HGT events affected gene families of potential importance for the acidophilic lifestyle of these AOA by playing a putative role in metal transport, detoxification or protection from stress. Four of the gene families share a common ancestor with acidophilic archaea (Table 2).

**Table 2:** List of horizontally acquired genes identified in the ‘*Nitrosotalea‐*specific core’ gene set.

Orthologous group	Predicted function	Genbank Accession for *Ca*. N. devanaterra	Present in other AOA	Database homologues (# used for phylogenies)^a^	Phylogenetically inferred gene exchange partner	Environment of gene exchange partner	Proteins detected in *Ca*. N. sinensis or *Ca*. N. okcheonensis cultures	Transcripts detected in *Ca*. N. bavarica metatranscriptome
OG2531	Divalent heavy‐metal cation transporter (zinc permease?)	CUR51883.1	No	55 (55)	Thermoplasmatales	Acidic soil	No	Yes
OG2888	Na^+^/H^+^solute symporter	CUR52062.1	No	999 (462)	Thermoplasmatales or, Crenarchaeota	Acidic hot springs and acid mine drainage	No	Yes
OG2912	acpD | FMN‐dependent NADH‐azoreductase	CUR52158.1	No	340 (183)	Ca. Div. Dependentiae,	Terrestrial aquifer sediment	No	No
OG2924	mntH | NRAMP family Mn^2+^/Fe^2+^ transporter	CUR51850.1	No	966 (477)	Woesarchaeota	Terrestrial aquifer sediment	No	Yes
OG2933	Coiled‐coil motif protein	CUR52192.1	No	11 (11)	Thermoplasmatales	Acid mine drainage	Yes	Yes
OG2943	FKBP‐type peptidyl‐prolyl cis‐trans isomerase	CUR51294.1	No	937 (513)	Methanosarcinales	Anaerobic environments	Yes	Yes
OG2932	Putative phage protein	CUR52193.1	No	7 (7)	Thermoplasmatales	Acid mine drainage	Yes	Yes
OG2113	Unknown (pentapeptide repeat containing protein)	CUR51439.1	Yes	1652 (1132)	*Sneathiella glossodoripedis*	Marine invertebrate symbiont	Yes	Yes

**a.** Orthologues identified in database were clustered at 95% amino acid identity prior to phylogenetic analysis.

### Expression of the *Candidatus* Nitrosotalea core genome

Proteomics and metatranscriptomics were used to assess which *Ca*. Nitrosotalea*‐*specific core genes are expressed. Proteomic analysis of *Ca*. Nitrosotalea strains Nd2 and CS, cultivated at optimal pH, identified 65% (1,227 proteins) and 13% (308 proteins) of all predicted proteins respectively (Supporting Information Tables SI.2.1 and SI.2.4). This confirmed expression of 62 of the 103 *Ca*. Nitrosotalea core gene families, four of which were horizontally acquired by a common ancestor of *Ca*. Nitrosotalea (Table 2 and Supporting Information Table SI.2.1). Metatranscriptomics data from the acidic fen, from which the *Ca*. Nitrosotalea strain SbT1 was assembled, confirmed transcription of 79 of the 103 *Ca*. Nitrosotalea‐specific core gene families, including seven of the gene families that were acquired via HGT, four of which were also identified through proteomics (Table 2 and Supporting Information Tables SI.2.1 and SI.2.5).

## Discussion

The four acidophilic thaumarchaeotal strains investigated in this study consistently form a monophyletic group branching as a sister clade to the Group I.1a Thaumarchaeota in phylogenetic trees based on *amoA* gene, 16S rRNA gene and two concatenated gene sets (Fig. 2 and Supporting Information Fig. S2c). ANI and AAI values clearly illustrate that the four strains are separate species. Due to their phylogeny and their high AAI values among each other, we assigned the four species to the *Ca*. genus Nitrosotalea and propose the following names for them: *Ca*. Nitrosotalea bavarica SbT1, *Ca*. Nitrosotalea okcheonensis CS and *Ca*. Nitrosotalea sinensis Nd2.

As *Ca*. Nitrosotalea occupies a unique low‐pH niche among cultivated Thaumarchaeota, the genomes of the four *Ca*. Nitrosotalea species were mined for gene families that they share to the exclusion of all other Thaumarchaeota, to identify candidate gene families that may be associated with their acidophilic lifestyle. In total 103 gene families were assigned to this ‘*Ca*. Nitrosotalea‐specific core’, almost half of which (50) are novel or too divergent for confident determination of function or origin. Another 46 families share homology with other Thaumarchaeota genes, despite being assigned to the *Ca*. Nitrosotalea specific core group. This is not surprising, given that a graph‐based orthologue definition will separate genes with homology if they are sufficiently different from one other, but strongly connected internally. It is possible that some of the gene families shared between all *Ca*. Nitrosotalea and other thaumarchaeotes facilitate life at low pH, with specific protein evolutionary adaptation, as previously observed for the ammonia monooxygenase protein (Macqueen and Gubry‐Rangin, 2016).

Careful phylogenetic analysis of the *Ca*. Nitrosotalea‐specific core gene families provided strong evidence that eight were affected by HGT events. For six of these gene families, *Ca*. Nitrosotalea genes clustered with homologues from non‐AOA archaeal phyla while two clustered with homologues from bacteria (Supporting Information Figs S3–S10). Interestingly, four HGT events (OGs 2531, 2888, 2932 and 2933) occurred with members of the Thermoplasmatales and in one case possibly also with acidophilic Crenarchaeota. The Thermoplasmatales is an order within the Euryarchaeota that comprises acidophiles growing preferentially below pH 2 and that also encompasses members of the genus *Picrophilus* growing at around pH 0, representing the most acidophilic microbes described (Huber and Stetter, 2006). For seven of the eight horizontally exchanged gene families, expression in culture and/or *in situ* could be confirmed (Table 2), suggesting their functional importance.

A previously published analysis of the *Ca*. N. devanaterra Nd1 genome identified 51 candidate genes of importance for its acidophilic lifestyle (Lehtovirta‐Morley *et al*., 2016). The analyses presented here now shows that a surprisingly low number (*n* = 10) of these genes are also present in all three newly determined *Ca*. Nitrosotalea genomes and absent in all other nonacidophilic AOA (Table 3 and Supporting Information Table SI.2.6). This is unlikely a result from lack of genome closure, as estimated genome completeness for all four *Ca*. Nitrosotalea was high (96.6%–100%). Therefore, the *Ca*. Nitrosotalea*‐*specific core genome likely lacks some of the typical mechanisms of pH homeostasis described in acidophiles (Baker‐Austin and Dopson, 2007). For example, the *kdp* potassium transporter (EC 3.6.3.12) of N*. devanaterra* is found in only two of the four *Ca*. Nitrosotalea genomes. This is unexpected as potassium is considered a critically important solute in extreme acidophiles, responsible for generating the reverse membrane potential and its absence implies that representatives of this genus either have a novel unrecognized mechanism for potassium uptake or use other cations to generate a reverse membrane potential. In contrast to a previous hypothesis (Lehtovirta‐Morley *et al*., 2016), the carbonic anhydrase (EC 4.2.1.1) of *Ca*. N. devanaterra Nd1 is not suitable for intracellular consumption of protons as it, like the respective homologues in other Thaumarchaeota, has an N‐terminal signal peptide, indicating an extracellular localization (Kerou *et al*., 2016). These γ‐class carbonic anhydrase (CA) homologs likely facilitate carbon transfer into the cell by converting bicarbonate to CO_2_, which can subsequently diffuse through the cell membrane. At an intracellular pH of 7, CO_2_ will be rehydrated to bicarbonate and used for carbon fixation. As members of the *Ca*. Nitrosotalea thrive in very low pH soils containing much more CO_2_ than bicarbonate, extracellular carbonic anhydrases are not necessary. Consistently, two of the four *Ca*. Nitrosotalea species (*Ca*. N. okcheonensis CS and *Ca*. N. sinensis Nd2) do not encode this enzyme.

**Table 3:** Re‐evaluation of *Ca*. N. devanaterra‐specific genes proposed to be involved in acidophily in Lehtovirta‐Morley *et al*. 2016.

Locus ID	Product	HGT	MT	NCS_Pr	Nd2_Pr
Genes present in the *Ca*. Nitrosotalea–specific core that were previously postulated as *Ca*. N. devanaterra‐specific genes involved in acidophily					
NDEV_0529	FKBP‐type peptidyl‐prolyl cis‐trans isomerase	X	X	X	
NDEV_0651	Coiled‐coil motif protein		X		
NDEV_0721	Protein of unknown function		X		X
NDEV_0771	Exported protein of unknown function		X		
NDEV_1085	NRAMP family Mn^2+^/Fe^2+^ transporter	X	X		
NDEV_1297	Na^+^/solute symporter	X	X		
NDEV_1333	Exported protein of unknown function		X		
NDEV_1368	Chromosome segregation ATPase‐like protein		X		X
NDEV_1562	Protein of unknown function				X
NDEV_1577	Membrane protein of unknown function				
Genes present in the *Ca*. Nitrosotalea–specific core that were previously postulated as *Ca*. N. devanaterra‐specific genes involved in acidophily and that have homologues in *Ca*. Nitrosocosmicus oleophilus MY3					
NDEV_1587	Na^+^/H^+^ exchanger		X		
NDEV_1999	Archaeal/V‐type ATP synthase subunit I		X		X
NDEV_2005	Archaeal/V‐type ATP synthase subunit F		X		X
NDEV_2006	Archaeal/V‐type ATP synthase subunit C		X	X	X

HGT: Acquired by HGT

MT: Detected in soil metatranscriptome

NCS_Pr: Detected in proteome of NCS

ND2_Pr: Detected in proteome of Nd2

In Lehtovirta *et al*., *Ca*. N. devanaterra‐specific genes that possessed homologues to other acidophilic microbes were considered as candidate genes involved in acidophily. In total, 51 genes were identified by that procedure. This table shows that only 10 of these genes are present in all four *Ca*. Nitrosotalea genomes and have no homologues in other non‐acidophilic thaumarchaeotes. In addition, four of the previously identified genes are present in all four *Ca*. Nitrosotalea genomes and in the AOA *Ca*. Nitrosocosmicus oleophilus MY3.

Interestingly, a specific subset of gene families thought to play a role in adaptation to low pH in *Ca*. N. devanaterra Nd1 are exclusively shared among AOA between the four *Ca*. Nitrosotalea genomes and *Ca*. Nitrosocosmicus oleophilus MY3, an AOA that can grow between pH 5.5 and 8.5 (Jung *et al*., 2014) (Table 3). For instance, all five genomes encode electroneutral CPA1‐type (cation/proton antiporter) Na^+^/H^+^ antiporters (TC 2.A.36) that were postulated to be involved in pH homeostasis in *Ca*. Nitrosotalea. In contrast, all neutrophilic AOA, including *Ca*. N. oleophilus MY3, possess electrogenic CPA2‐type Na^+^/H^+^ exchangers (TC 2.A.37) (Padan *et al*., 2005) that are absent in genus *Ca*. Nitrosotalea (OG0030, OG0824). It has previously been demonstrated that CPA1‐type transporters export protons and are downregulated at alkaline pH (Călinescu *et al*., 2014), while CPA2‐type transporters are downregulated at acidic pH (Alkoby *et al*., 2014), although it is not clear whether this distinction applies to all CPA1‐ and CPA2‐type exchangers. Likewise, subunits of the membrane‐bound domain and central and peripheral stalks of the A‐type ATP synthase of *Ca*. Nitrosotalea and *Ca*. N. oleophilus MY3 (EC 3.6.3.14) were dissimilar (< 30% AA identity) to other AOA. The functional implications of this divergence are currently unknown. In contrast, the cytoplasmic domain (A_1_) (*atpAB*) of the ATP synthase is conserved in all AOA, including *Ca*. Nitrosotalea genomes. The direction of proton transport by A‐type ATP synthase is reversible (Grüber *et al*., 2014), and, thus, the modified ATP synthase may be involved in proton extrusion in the genus *Ca*. Nitrosotalea. This would necessitate a dual role in ATP synthesis and proton export since the A‐type ATP synthase is the only ATP synthase encoded in the genomes of *Ca*. Nitrosotalea.

### *Ca.* Nitrosotalea core gene families acquired through horizontal gene transfer

Five of the *Ca*. Nitrosotalea‐specific core gene families affected by HGT might play important roles in the acidophilic lifestyle of these Thaumarchaeota (Table 2), suggesting that horizontal gene acquisition was important for their adaptation to low pH environments. First, one of the gene families exchanged with members of the Thermoplasmatales is a Na^+^/solute symporter (OG2888) (Lehtovirta‐Morley *et al*., 2016) and is present in many bacterial and archaeal acidophiles. Characterized members of the Na^+^/solute symporter family (TC 2.A.21) take up a wide range of organic solutes, including amino acids, sugars and monocarboxylates and dicarboxylates (Jung, 2002; Groeneveld *et al*., 2010). Amino acid alignment suggests that the Na^+^/solute symporters of *Ca*. Nitrosotalea lack the sodium binding site (data not shown) and the phylogenetic placement of the four *Ca*. Nitrosotalea species transporters with characterized homologues consistently recovers a robust relationship to *mctP* of *R. leguminosarum*, a proton‐coupled monocarboxylic acid symporter (Supporting Information Fig. S11) (Hosie *et al*., 2002; Jung, 2002). This implies proton‐ rather than sodium‐coupled symport. Uptake of organic compounds seems paradoxical because the three cultivated *Ca*. Nitrosotalea strains grow autotrophically in inorganic media. However, there is evidence for stimulation of *Ca*. Nitrosotalea growth by some organic acids, for example, oxaloacetate (Lehtovirta‐Morley *et al*., 2014). While we can only speculate on the function of this protein, its conservation in *Ca*. Nitrosotalea core genome, its consistent presence in other archaeal acidophiles, and its absence from all other AOA makes it a strong candidate for future characterization and determination of the substrate specificity together with its role in acidophily.

Two more genes of the horizontally transferred *Ca*. Nitrosotalea*‐*specific core encode metal transporters (OG2531 and OG2924). While OG2531 is a member of the Zinc‐Iron Permease (ZIP) family and can be annotated with high confidence as a Zn^2+^ importer, OG2924 is a member of the divalent cation transporter NRAMP (TC 2.A.55) family found in many acidophiles for which substrate predictions are not possible without experiments. The gene families OG2531 and OG2924 were horizontally exchanged with members of the Thermoplasmatales and Woesearchaeota respectively. We postulate that these metal transporters provide adaptation for metal uptake under low pH conditions, where the bioavailability of metals is strongly increased (Violante *et al*., 2010) and transporters with different properties (e.g., a lower affinity) might be beneficial. Interestingly, all other genome‐sequenced AOA also encode a ZIP transporter (not closely related and likely replaced by the laterally acquired ZIP in *Ca*. Nitrosotalea, data not shown). In most AOA, this transporter is located immediately adjacent to the multicopper oxidase 1 (MCO 1), which has recently been hypothesized as an interesting candidate for thaumarchaeotal hydroxylamine oxidation (Kerou *et al*., 2016). Interestingly, however, MCO 1 is absent from all four *Ca*. Nitrosotalea species.

A fourth gene that has been laterally exchanged between *Ca*. Nitrosotalea and other archaea, belonging to the Methanosarcinales, is a FKBP‐type peptidyl‐prolyl cis‐trans isomerase gene (OG2943) encoding a folding chaperone for proteins containing proline residues. While classified within the *Ca*. Nitrosotalea‐specific core, distantly related FKBP‐type peptidyl‐prolyl cis‐trans isomerases are also found in neutrophilic AOA, indicating that not all folding chaperones are confined to AOA with an acidophilic lifestyle. Although homologues of OG2943 have not been linked specifically to acidophily in other organisms, chaperones in general are prevalent in acidophilic genomes and upregulated during pH down‐shift (Baker‐Austin and Dopson, 2007).

Finally, a FMN‐dependent NADH‐azoreductase (EC 1.7.1.6; OG2912) that has been exchanged with members of the recently proposed bacterial candidate phylum ‘Dependentiae’ (Yeoh *et al*., 2016) is present in all analysed *Ca*. Nitrosotalea species and may function in detoxification of reactive nitrogen compounds (Nakanishi *et al*., 2001; Ryan *et al*., 2010). Diazo compounds may be formed by reaction between amine side groups with reactive nitrogen (e.g., nitrous acid, hydroxylamine), which is particularly important at low pH, although they have been reported to occur rarely naturally (Nawrat and Moody, 2011).

### Species‐specific features of individual *Ca.* Nitrosotalea genomes

Several unexpected species‐specific genes were observed in the newly determined *Ca*. Nitrosotalea genomes. For example, *Ca*. Nitrosotalea bavarica SbT1 harbours an archaeal (type III) RuBisCO (SCTHAUMv1_33063) implicated in CO_2_ fixation, although other key Calvin cycle genes (e.g., phosphoribulokinase) are missing. This gene may function in the AMP salvage pathway as described for hot spring Thaumarchaeota (Beam *et al*., 2014), particularly as another gene of the same pathway, encoding an AMP phosphorylase (SCTHAUMv1_33062), is located adjacent to the RuBisCO‐encoding gene. In other archaea, for example, *Pyrococcus furiosus*, excess AMP can be generated through saccharolytic activity, but *Ca*. N. bavarica SbT1 contains no ADP‐dependent phosphofructokinase homologue or other recognisable ADP‐dependent sugar kinases. As proposed for *Thermococcus kodakaraensis* (Sato *et al*., 2007), AMP may be produced instead through degradation of 5‐phosphoribosyl 1‐pyrophosphate (PRPP) by adenine phosphoribosyltransferase (SCTHAUM_10121), which is also encoded in other Thaumarchaeota. PRPP is produced in Thaumarchaeota by ribose‐phosphate pyrophosphokinase (SCTHAUM_90122), as part of nucleotide biosynthesis and can also spontaneously break down into ribose‐1,5‐BP. This could then be converted into ribulose 1,5‐bisphosphate by ribose 1,5‐bisphosphate isomerase of *Ca*. N. bavarica SbT1 (SCTHAUM_70401), providing a substrate for the RuBisCO, as demonstrated in the methanogenic archaeon *M. jannaschii* (Finn and Robert Tabita, 2004), linking the pentose phosphate pathway and gluconeogenesis. Although *Ca*. N. bavarica SbT1 has the homologue of ribose‐1,5‐bisphosphate isomerase found in *M. jannaschii* (Mj0601) (Finn and Robert Tabita, 2004), related proteins are also implicated in thiazole metabolism (Hwang *et al*., 2014), and the function of the protein and the existence of this pathway in *Ca*. N. bavarica SbT1 needs to be verified experimentally, when a cultured member of this species becomes available.

Ni‐Fe hydrogenase, gas vacuoles, genes for flagellar motility and chemotaxis and phosphate utilization genes are also encoded by some but not all genomes of *Ca*. Nitrosotalea (Supporting Information Table SI.2.7), providing testable hypotheses for adaptations of *Ca*. Nitrosotalea strains to factors other than pH.

Interestingly, the *Ca*. N. okcheonensis CS genome has two *amoA* gene copies in contrast to all previously genome‐sequenced AOA, which have a single *amoA* gene. One copy (NCS_11555) was found in the canonical arrangement *amoAxCB*, as in other *Ca*. Nitrosotalea genomes, and is transcribed during growth in batch culture (Supporting Information 1). The second copy (NCS_11033), which is located >400 kb upstream from the *amoAxCB* gene cluster, shares 95.5% DNA similarity with the first, but was not transcriptionally active under standard growth conditions (Supporting Information Fig. S12). The local genomic region surrounding each *amoA* gene was confirmed by PCR amplification using primers designed to hybridize to adjacent ORFs. Multiple copies of the *amoCAB* operon can be found in AOB, and additional isolated copies of *amoC* can be found in both AOA and AOB (Spang *et al*., 2012). In addition, two divergent copies of *amoB* were recently reported in the marine AOA *Ca*. N. piranensis D3C (Bayer *et al*., 2016). The isolated *amoC* gene in *Nitrosomonas europaea* is not transcribed during growth, but only during a poststarvation stress response (Berube and Stahl, 2012). It is difficult to predict if, and under which conditions, the genomically isolated *amoA* gene of *Ca*. N. okcheonensis CS is transcribed, but its existence has immediate implications for molecular studies of AOA in the environment. The *amoA* gene is the most widely used marker for determining AOA and AOB diversity and abundance in environmental samples and the existence of two nonidentical copies of this gene may lead to overestimation of AOA diversity and abundance, given the common assumption of one *amoA* gene per AOA genome (Trias *et al*., 2012).

In conclusion, comparative genomics of four *Ca*. Nitrosotalea species enabled identification of a core set of gene families for this genus encompassing 103 gene families. Expression of the majority of these genes families was confirmed by proteomics under laboratory conditions and metatranscriptomics in an incubation experiment with acidic peat soil. Although the four analysed *Ca*. Nitrosotalea species all thrive at low pH, their genomic core excluded many gene families that were previously proposed to represent adaptations of *Ca*. N. devanaterra Nd1 to acidic environments (Lehtovirta‐Morley *et al*., 2016). Interestingly, some of the core genes with an inferred function for acidophily were clearly acquired by *Ca*. Nitrosotalea via horizontal gene transfer from other microbial groups, including the acidophilic Thermoplasmatales, demonstrating that adaptation of *Ca*. Nitrosotalea members to their low pH environment was facilitated by implementation of mechanisms having evolved in other microbes of these systems. It will be interesting to explore whether similar mechanisms for pH adaptation are also used by other (non‐*Ca*. Nitrosotalea) thaumarchaeotal lineages that are abundant in acidic soils (Gubry‐Rangin *et al*., 2011), but for which no genome sequences are yet available.

## Experimental procedures

### Thaumarchaeotal genomes

Genomes of four members of the genus *Ca*. Nitrosotalea, abundant in acidic soils, were compared in this study. Three of the genomes originated from pure cultures or enrichments: *Ca*. Nitrosotalea devanaterra Nd1 was isolated from a Scottish agricultural soil (pH 4.5) and its complete genome was recently sequenced (Lehtovirta‐Morley *et al*., 2016), *Ca*. Nitrosotalea sinensis Nd2 was isolated from a Chinese acidic paddy soil (pH 4.7) (Lehtovirta‐Morley *et al*., 2014) and *Ca*. Nitrosotalea okcheonensis CS was enriched from a Korean soil (pH 3.2) contaminated with acid mine drainage water (Jung *et al*., 2014). Details of the cultivation, DNA extraction (Bramwell *et al*., 1995), genome sequencing and assembly are given in Supporting Information. In contrast, the genome of *Ca*. Nitrosotalea bavarica strain SbT1 was assembled and binned (Albertsen *et al*., 2013) from a metagenomic dataset of the minerotrophic fen Schlöppnerbrunnen II (50°07′54.8″ N, 11°52′51.8″ E, 713 m above sea level, typical pH 4–5), located in the Fichtelgebirge Mountains in north‐eastern Bavaria, Germany (Herrmann *et al*., 2012; Pester *et al*., 2012; Hausmann *et al*., 2016). For further details on this metagenomic experiment see Supporting Information 1 and Hausmann *et al*. (2016).

In addition to the four *Ca*. Nitrosotalea genomes, 19 other thaumarchaeotal genomes were compared (Table 1). For all 23 genomes, genome composition completeness was estimated using thaumarchaeotal‐based arCOG markers (Rinke *et al*., 2014; Supporting Information Table SI.2.8) and CheckM (Parks *et al*., 2015), while genus and species assignments were evaluated using ANI and AAI (Richter and Rosselló‐Móra, 2009; Konstantinidis and Tiedje, 2005) (see details in Supporting Information 1).

### Comparative genomics

The core and flexible genomes of *Ca*. Nitrosotalea were identified using the MicroScope platform for annotation (Vallenet *et al*., 2009) and OrthoMCL (Li *et al*., 2003), which uses a Markov Cluster algorithm to assign coding sequences to orthologous groups based on all‐against‐all BLASTp (Supporting Information Table SI.2.9). The core genome for AOA was defined as all orthologue groups for which all AOA had at least one coding sequence. Accordingly, the core genome of specific genera of AOA (e.g., *Ca*. Nitrosotalea) was defined as all orthologue groups for which all members had at least one coding sequence and no other AOA possessed a corresponding orthologue. The flexible genome was defined as orthologue groups which contained coding sequences from multiple AOA, but not from all AOA. Theoretical core genome and pangenome sizes were estimated (Contreras‐Moreira & Vinuesa, 2013; Supporting Information 1), while genomic synteny was calculated between all *Ca*. Nitrosotalea genomes (Kurtz *et al*., 2004; Supporting Information 1 and Fig. S13).

### Origin of individual gene families comprising the *Candidatus* Nitrosotalea core genome

Gene families that comprised the core genome of *Ca*. Nitrosotalea were examined to identify possible origin scenarios. Each gene was used as a query in a blastp search against the Genbank nr protein database using default parameters, except returning up to 1,000 subjects for each query. All hits that matched at least one query over 70% of its length and with >30% identity were collected as ‘database homologues’ (Table 1). From this set of database homologues, usearch (Edgar, 2010) was used to cluster database entries at 95% amino acid identity. Centroids were aligned using mafft (Katoh and Standley, 2013) and preliminary trees were constructed using FastTree (Price *et al*., 2009). Gene families were classified as ‘*Ca*. Nitrosotalea‐specific’ if no database entries outside the known *Ca*. Nitrosotalea was identified with blastp. Gene families were classified as ‘*Ca*. Nitrosotalea‐specific with low AA‐identity to non‐AOA’ if there were only one to three database matches at low identity (30%–45% amino acid identity). The remainder of the gene families of the *Ca*. Nitrosotalea core genome were examined phylogenetically. If a gene family formed a clade with other Thaumarchaeota, it was assumed to be a divergent form of the homologue in other Thaumarchaeota. Phylogenetic trees were recalculated using RAxML (Stamatakis, 2015) for the remaining gene families, to verify the relationship of the *Ca*. Nitrosotalea gene family with its nearest phylogenetic neighbour(s), which was inferred to be the donor lineage of that gene family to a *Ca*. Nitrosotalea common ancestor.

### Phylogenomic and phylogenetic approaches

Two independent phylogenomic approaches were implemented, maximum‐likelihood (Stamatakis, 2015) on 198 phylogenetically congruent single‐copy marker genes (Fig. 2) or Bayesian‐likelihood (Lartillot *et al*., 2009) on 34 universal marker genes subset identified with CheckM (Supporting Information Fig. S2c) (Parks *et al*., 2015). In addition, the 16S rRNA (Supporting Information Fig. S2a) and *amoA* (Supporting Information Fig. S2b) gene Bayesian phylogenies were performed as described in Gubry‐Rangin *et al*. (2015). More details on these approaches can be found in Supporting Information 1.

### Experimental validation of *in silico* predictions

To confirm the presence of two *amoA* genes (ORF11033 and 11555) in *Ca*. N. okcheonensis CS and generate qPCR standards for expression analysis, PCR primers were designed that hybridized at positions within adjacent predicted ORFs (11032/11034 and 11554/11556), with a further set of primers that hybridized within ORF11033 and ORF11555 to amplify mRNA transcripts (Supporting Information Table SI.2.10). Total RNA was extracted from cells harvested from 500 ml of an exponentially growing culture using the RNeasy Mini Kit (Qiagen, Germany) and cDNA synthesized using the SuperScript First Strand synthesis system (Invitrogen, San Diego, CA) according to manufacturer's instructions. Concentrations of RNA and cDNA were determined using an ND‐1000 spectrophotometer (NanoDrop Technologies, Wilmington, DE). Quantitative real‐time PCR experiments were carried out using a MiniOpticon real‐time PCR detection system (Bio‐Rad Laboratories, Hercules, CA) and Opticon Monitor Software version 3.1 (Bio‐Rad Laboratories, Hercules, CA). Thermal cycling parameters were 15 min at 95°C, followed by 40 cycles at 95°C for 20 s, 55°C for 20 s and 72°C for 20 s, with readings recorded after each cycle. A control 16S rRNA gene assay was also performed as described previously (Jung *et al*., 2014). PCR efficiency was 87%–95% with *r* ^2^ values ≥0.99 for all assays.

Genomic *in silico* predictions in *Ca*. Nitrosotalea strains were validated by analysing the proteomic profiles of two of the three cultured thaumarchaeotal strains after growth under optimal conditions (pH = 5.3 and 5.0 for *Ca*. N. sinensis Nd2 and *Ca*. N. okcheonensis CS respectively). Cells from seven replicate cultures (1,000 ml and 500 ml each for strains Nd2 and CS respectively) were harvested individually by filtration and stored at −80°C upon protein extraction with denaturing SDS buffer and proteomic analysis by LC‐MS (see Supporting Information 1). Genomic *in silico* predictions in *Ca*. Nitrosotalea strains were validated by metatranscriptomics analysis of samples from anoxic peat soil microcosms with or without amendments of several organic compounds (see Hausmann *et al*., 2016 and Supporting Information 1).

## Supporting information

Additional Supporting Information may be found in the online version of this article at the publisher's web‐site:

**Fig. S1.** Differential coverage plot comparing a metagenome obtained from native fen soil and a metagenome obtained from the heavy fractions of the same soil after SIP. Only scaffolds larger than 10 knt are shown. Scaffolds binned to *Ca*. N. bavarica SbT1 are marked by black borders. Scaffolds with no reads mapped from untreated or SIP metagenomes are drawn directly on the *x*‐ and *y*‐axis respectively.
**Fig. S2.** Bayesian phylogenetic trees of the (a) 16S rRNA and (b) *amoA* genes of the 23 AOA strains used in this study with posterior values > 0.5 indicated for each branch. (c) Phylobayes‐constructed phylogenetic relationship of the four *Ca*. Nitrosotalea species with other genome‐sequenced AOA based on a set of concatenated universal marker genes identified with CheckM. Bayesian posterior support of internal branches is shown. The outgroup consists of Lokiarchaeota, *Thermophilum*, Bathyarchaeota, Korarchaeota, *Thermococcus* and *Caldiarchaeum*.
**Fig. S3.** Maximum‐likelihood phylogenetic tree of an exported protein of unknown function (OG2113) that is *Ca*. Nitrosotalea‐specific among Thaumarchaeota using a graph‐based orthologue definition, despite the fact that distant homologues are found in other Thaumarchaeota. Thaumarchaeotal homologues and homologues found distributed among other archaeal and bacterial lineages are displayed. Taxa are coloured according to phylum and accession numbers are provided. Genes from *Ca*. Nitrosotalea are highlighted. The complete sequence set was identified using *Ca*. Nitrosotalea amino acid sequences as individual queries for blastp searches against the NCBI nr database. Hits were screened for amino acid identity > 30% over 70% of the length of any single *Ca*. Nitrosotalea query ortholog. The whole dataset consisted of 6 *Ca*. Nitrosotalea and 1136 database hits. The four closest phylogenetic neighbours are shown here and the outgroup consists of 1132 additional database hits. The relationship of the ingroup with respect to individual outgroup clades remains unresolved. Proportional bootstrap support > 0.5 is shown.
**Fig. S4.** Maximum‐likelihood phylogenetic tree of a putative divalent heavy‐metal cations transporter (OG2531) that is *Ca*. Nitrosotalea‐specific among Thaumarchaeota but also found distributed among other nonthaumarchaeotal lineages. Taxa are coloured according to phylum and accession numbers are provided. Genes from *Ca*. Nitrosotalea are highlighted. The complete sequence set was identified using *Ca*. Nitrosotalea amino acid sequences as queries for blastp searches against the NCBI nr database. Hits were screened for amino acid identity > 30% over 70% of the length of any single *Ca*. Nitrosotalea query ortholog. The whole dataset consisted of four *Ca*. Nitrosotalea and 51 database hits. The five closest phylogenetic neighbours are shown and the outgroup consists of 46 additional database hits. The relationship of the ingroup with respect to individual outgroup clades remains unresolved. Proportional bootstrap support > 0.5 is shown.
**Fig. S5.** Maximum‐likelihood phylogenetic tree of an Na+/solute symporter (OG2888) that is *Ca*. Nitrosotalea‐specific among Thaumarchaeota but also found distributed among other nonthaumarchaeotal lineages. Taxa are coloured according to phylum and accession numbers are provided. Genes from *Ca*. Nitrosotalea are highlighted. The complete sequence set was identified using *Ca*. Nitrosotalea amino acid sequences as queries for blastp searches against the NCBI nr database. Hits were screened for amino acid identity > 30% over 70% of the length of any single *Ca*. Nitrosotalea query ortholog. The whole dataset consisted of four *Ca*. Nitrosotalea and 462 database hits. The 32 closest phylogenetic neighbours are shown and the outgroup consists of 430 additional database hits. The relationship of the ingroup with respect to individual outgroup clades remains unresolved. Proportional bootstrap support > 0.5 is shown.
**Fig. S6.** Maximum‐likelihood phylogenetic tree of an FMN‐dependent NADH‐azoreductase (OG2912) that is *Ca*. Nitrosotalea‐specific among Thaumarchaeota but also found distributed among other nonthaumarchaeotal lineages. Taxa are coloured according to phylum and accession numbers are provided. Genes from *Ca*. Nitrosotalea are highlighted. The complete sequence set was identified using *Ca*. Nitrosotalea amino acid sequences as queries for blastp searches against the NCBI nr database. Hits were screened for amino acid identity > 30% over 70% of the length of any single *Ca*. Nitrosotalea query ortholog. The whole dataset consisted of four *Ca*. Nitrosotalea and 201 database hits. The 14 closest phylogenetic neighbours are shown and the outgroup consists of 183 additional database hits. The relationship of the ingroup with respect to individual outgroup clades remains unresolved. Proportional bootstrap support > 0.5 is shown.
**Fig. S7.** Maximum‐likelihood phylogenetic tree of an NRAMP family Mn2+/Fe2+ transporter (OG2924) that is *Ca*. Nitrosotalea‐specific among Thaumarchaeota but also found distributed among other nonthaumarchaeotal lineages. Taxa are coloured according to phylum and accession numbers are provided. Genes from *Ca*. Nitrosotalea are highlighted. The complete sequence set was identified using *Ca*. Nitrosotalea amino acid sequences as queries for blastp searches against the NCBI nr database. Hits were screened for amino acid identity > 30% over 70% of the length of any single *Ca*. Nitrosotalea query ortholog. The whole dataset consisted of four *Ca*. Nitrosotalea and 477 database hits. The closest phylogenetic neighbour is shown and the outgroup consists of 476 additional database hits. The relationship of the ingroup with respect to individual outgroup clades remains unresolved. Proportional bootstrap support > 0.5 is shown.
**Fig. S8.** Maximum‐likelihood phylogenetic tree of a putative phage protein (OG2932) that is *Ca*. Nitrosotalea‐specific among Thaumarchaeota but also found distributed among other nonthaumarchaeotal lineages. Taxa are coloured according to phylum and accession numbers are provided. Genes from *Ca*. Nitrosotalea are highlighted. The complete sequence set was identified using *Ca*. Nitrosotalea amino acid sequences as queries for blastp searches against the NCBI nr database. Hits were screened for amino acid identity > 30% over 70% of the length of any single *Ca*. Nitrosotalea query ortholog. The whole dataset consisted of four *Ca*. Nitrosotalea and seven database hits. All eleven taxa are shown and the tree is midpoint‐rooted. Proportional bootstrap support > 0.5 is shown.
**Fig. S9.** Maximum‐likelihood phylogenetic tree of a coiled‐coil motif protein of unknown function (OG2933) that is *Ca*. Nitrosotalea‐specific among Thaumarchaeota but also found distributed among other nonthaumarchaeotal lineages. Taxa are coloured according to phylum and accession numbers are provided. Genes from *Ca*. Nitrosotalea are highlighted. The complete sequence set was identified using *Ca*. Nitrosotalea amino acid sequences as queries for blastp searches against the NCBI nr database. Hits were screened for amino acid identity> 30% over 70% of the length of any single *Ca*. Nitrosotalea query ortholog. The whole dataset consisted of four *Ca*. Nitrosotalea and 11 database hits. All eleven taxa are shown and the tree is midpoint‐rooted. Proportional bootstrap support > 0.5 is shown.
**Fig. S10.** Maximum‐likelihood phylogenetic tree of an FKBP‐type peptidyl‐prolyl cis‐trans isomerase (OG2943) that is *Ca*. Nitrosotalea‐specific among Thaumarchaeota but also found distributed among other nonthaumarchaeotal lineages. Taxa are coloured according to phylum and accession numbers are provided. Genes from *Ca*. Nitrosotalea are highlighted. The complete sequence set was identified using *Ca*. Nitrosotalea amino acid sequences as queries for blastp searches against the NCBI nr database. Hits were screened for amino acid identity> 30% over 70% of the length of any single *Ca*. Nitrosotalea query ortholog. The whole dataset consisted of four *Ca*. Nitrosotalea and 513 database hits. The nine closest phylogenetic neighbour is shown here and the outgroup consists of 504 additional database hits. The relationship of the ingroup with respect to individual outgroup clades remains unresolved. Proportional bootstrap support > 0.5 is shown.
**Fig. S11.** Maximum‐likelihood phylogenetic tree of Na^+^/solute transporters based on sequences available in Jung (2002) plus the four *Ca*. Nitrosotalea sequences. Bootstrap values above 80% are indicated. Proteins from organisms shown in bold have been biochemically characterized. Accession numbers are provided in brackets.
**Fig. S12.** Transcript abundance of two *amoA* genes (ORF 11033 and 11555) and 16S rRNA in total RNA extracts from an exponentially growing culture of *Ca*. N. okcheonensis CS. Two different RT‐qPCR assays were used for each *amoA* gene. Error bars are the standard deviation of three replicates.
**Fig. S13.** Mummer plots between *Ca*. Nitrosotalea genomes. Genomic coordinates are given in megabases (Mb). ‘Forward’ alignments are shown in blue. Reverse‐complement alignments are shown in red.
**Fig. S14.** Abundance of thaumarchaeotal *amoA* genes in DNA extractions from anoxic Schlöppnerbrunnen peat soil microcosms incubated with different substrates (for details see Hausmann *et al*., 2016). No increase in, but persistence of thaumarchaeotal *amoA* genes was observed after 50 days of incubation in all treatments. Bar height corresponds to the mean and error bars are the standard deviation of three replicate measurements.
**Fig. S15.** Theoretical core genome (A, B) and theoretical pangenome (C, D) sizes of 23 AOA (panel A, C) and four *Ca*. Nitrosotalea (B, D) strains. Random sampling was performed 10 times and the exponential models described in Tettelin *et al*. (2005) (red) and Willenbrock *et al*. (2007) (blue) were used to predict the size of core genomes (A and B) and pangenomes (C and D) extrapolated to infinite genomes sampled.
