# Supplementary material for: Ammonia‐oxidising archaea living at low pH: Insights from comparative genomics
Source: Environ Microbiol. 2017 Dec 4;19(12):4939–52. doi: 10.1111/1462-2920.13971 (PMC5767755; doi:10.1111/1462-2920.13971)
Supplement: Supplementary file 1 — Fig. S1. Differential coverage plot comparing a metagenome obtained from native fen soil and a metagenome obtained from the heavy fractions of the same soil after SIP. Only scaffolds larger than 10 knt are shown. Scaffolds binned to Ca. N. bavarica SbT1 are marked by black borders. Scaffolds with no reads mapped from untreated or SIP metagenomes are drawn directly on the x‐ and y‐axis respectively. Fig. S2. Bayesian phylogenetic trees of the (a) 16S rRNA and (b) amoA genes of the 23 AOA strains used in this study with posterior values > 0.5 indicated for each branch. (c) Phylobayes‐constructed phylogenetic relationship of the four Ca. Nitrosotalea species with other genome‐sequenced AOA based on a set of concatenated universal marker genes identified with CheckM. Bayesian posterior support of internal branches is shown. The outgroup consists of Lokiarchaeota, Thermophilum, Bathyarchaeota, Korarchaeota, Thermococcus and Caldiarchaeum. Fig. S3. Maximum‐likelihood phylogenetic tree of an exported protein of unknown function (OG2113) that is Ca. Nitrosotalea‐specific among Thaumarchaeota using a graph‐based orthologue definition, despite the fact that distant homologues are found in other Thaumarchaeota. Thaumarchaeotal homologues and homologues found distributed among other archaeal and bacterial lineages are displayed. Taxa are coloured according to phylum and accession numbers are provided. Genes from Ca. Nitrosotalea are highlighted. The complete sequence set was identified using Ca. Nitrosotalea amino acid sequences as individual queries for blastp searches against the NCBI nr database. Hits were screened for amino acid identity > 30% over 70% of the length of any single Ca. Nitrosotalea query ortholog. The whole dataset consisted of 6 Ca. Nitrosotalea and 1136 database hits. The four closest phylogenetic neighbours are shown here and the outgroup consists of 1132 additional database hits. The relationship of the ingroup with respect to individual outgroup clades r [file EMI-19-4939-s001.pdf]

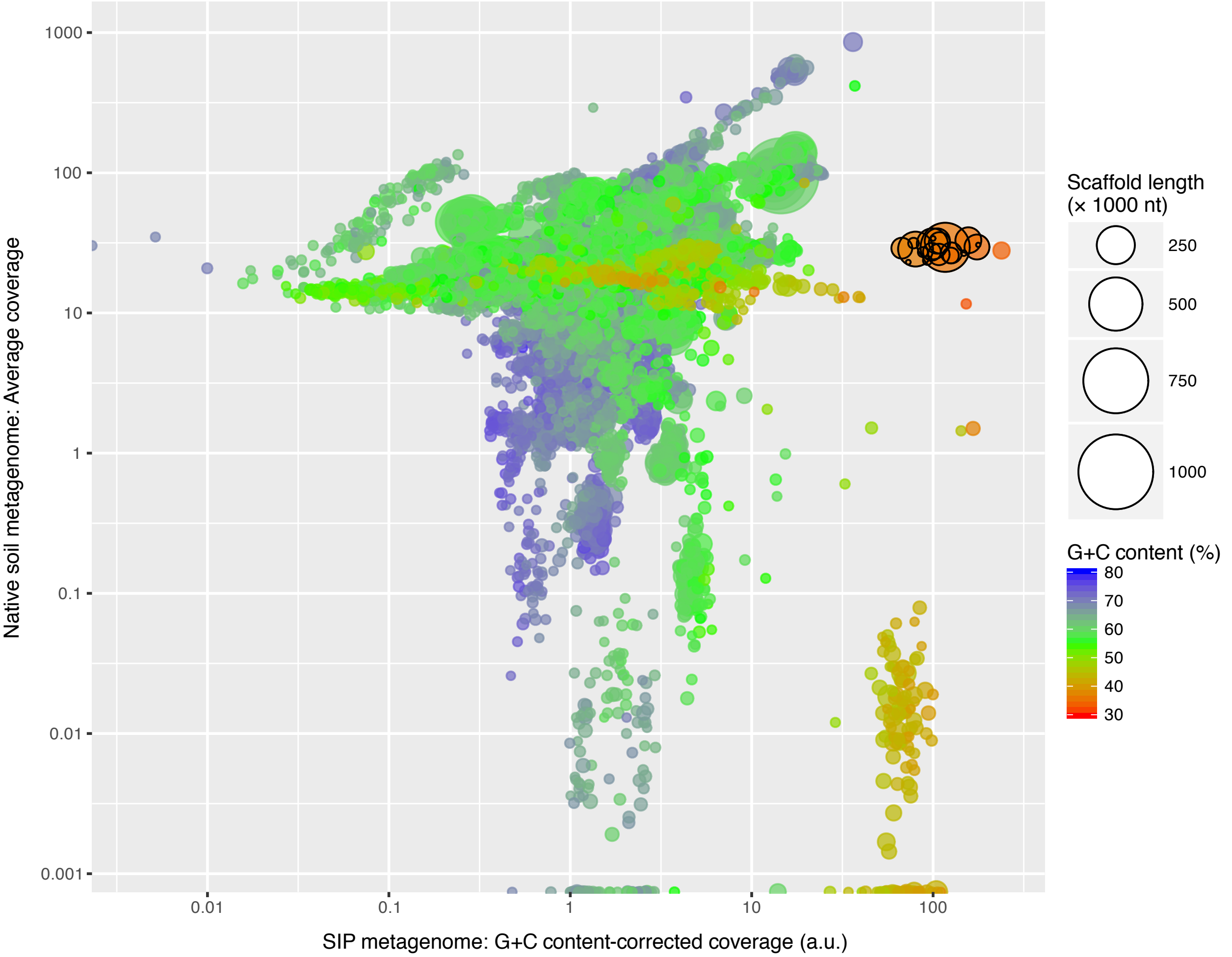

Figure S1. Differential coverage plot comparing a metagenome obtained from native fen soil and a metagenome obtained from the heavy fractions of the same soil after SIP. Only scaffolds larger than 10 knt are shown. Scaffolds binned to *Ca.* N. bavarica SbT1 are marked by black borders. Scaffolds with no reads mapped from native or SIP metagenomes are drawn directly on the x- and y-axis, respectively

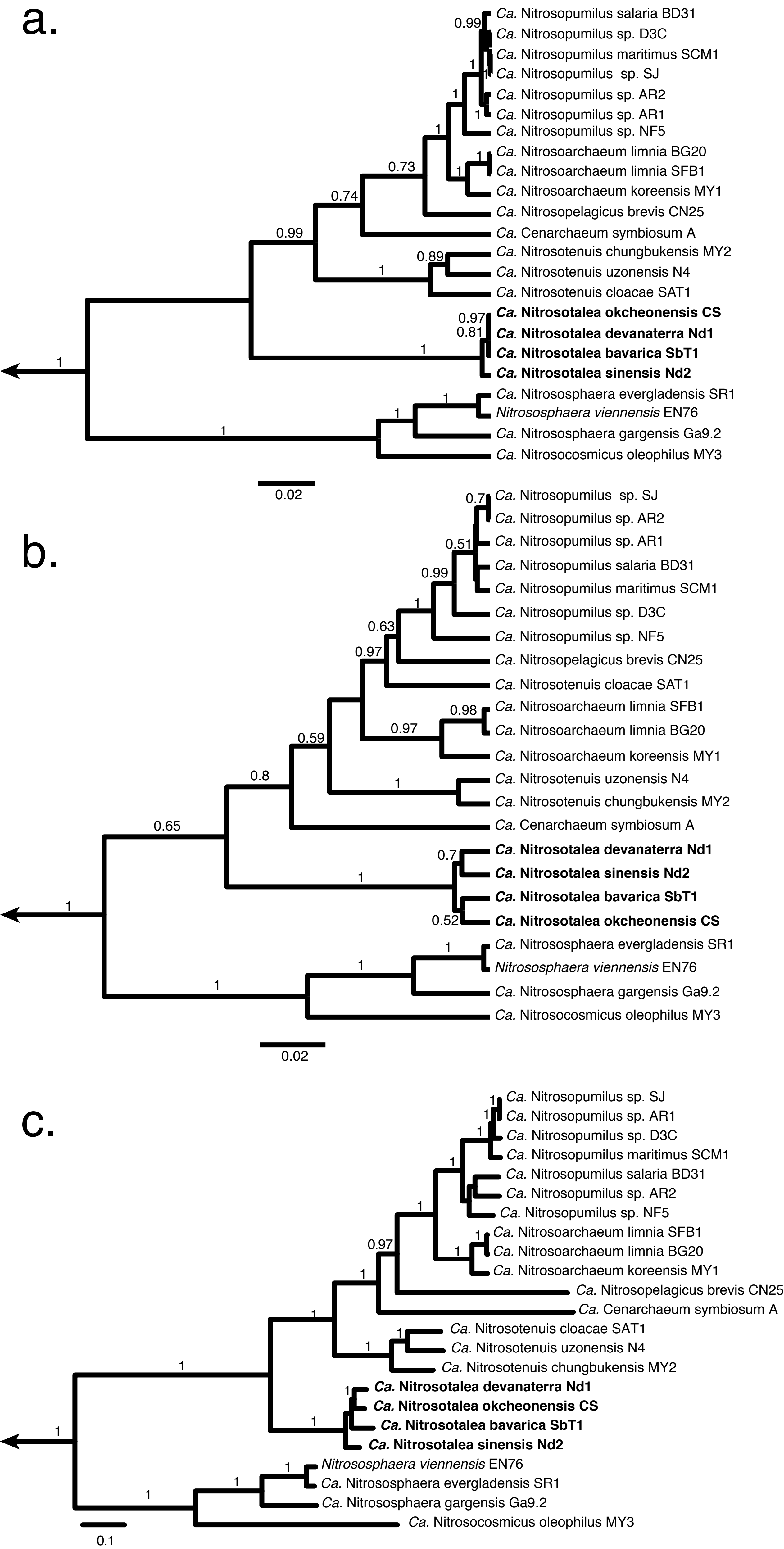

Figure S2. Bayesian phylogenetic trees of the **(a)** 16S rRNA and **(b)** *amoA* genes of the 23 AOA strains used in this study with posterior values >0.5 indicated for each branch. **(c)** Phylobayes-constructed phylogenetic relationship of the four *Ca.* Nitrosotalea species with other genome-sequenced AOA based on a set of concatenated universal marker genes identified with CheckM. Bayesian posterior support of internal branches is shown. The outgroup consists of *Lokiarchaeota*, *Thermophilum*, *Bathyarchaeota*, *Korarchaeota*, *Thermococcus* and *Caldiarchaeum*.

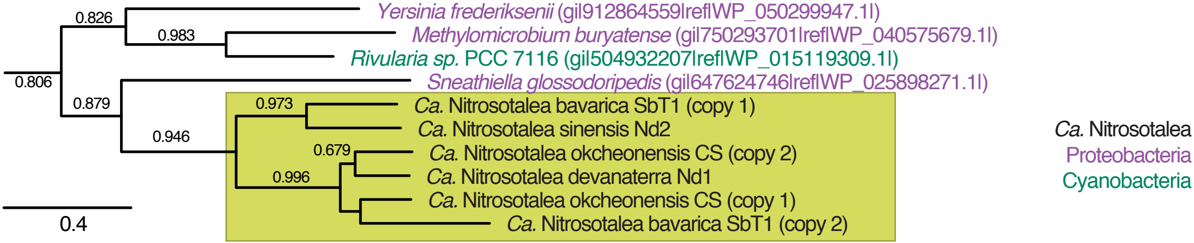

Figure S3. Maximum-likelihood phylogenetic tree of an exported protein of unknown function (OG2113) that is *Ca.* Nitrosotalea-specific among Thaumarchaeota using a graph-based orthologue definition, despite the fact that distant homologues are found in other Thaumarchaeota. Thaumarchaeotal homologues and homologues found distributed among other archaeal and bacterial lineages are displayed. Taxa are coloured according to phylum and accession numbers are provided. Genes from *Ca.* Nitrosotalea are highlighted. The complete sequence set was identified using *Ca.* Nitrosotalea amino acid sequences as individual queries for blastp searches against the NCBI nr database. Hits were screened for amino acid identity >30% over 70% of the length of any single *Ca.* Nitrosotalea query ortholog. The whole dataset consisted of 6 *Ca.* Nitrosotalea and 1,136 database hits. The four closest phylogenetic neighbours are shown here and the outgroup consists of 1,132 additional database hits. The relationship of the ingroup with respect to individual outgroup clades remains unresolved. Proportional bootstrap support >0.5 is shown.

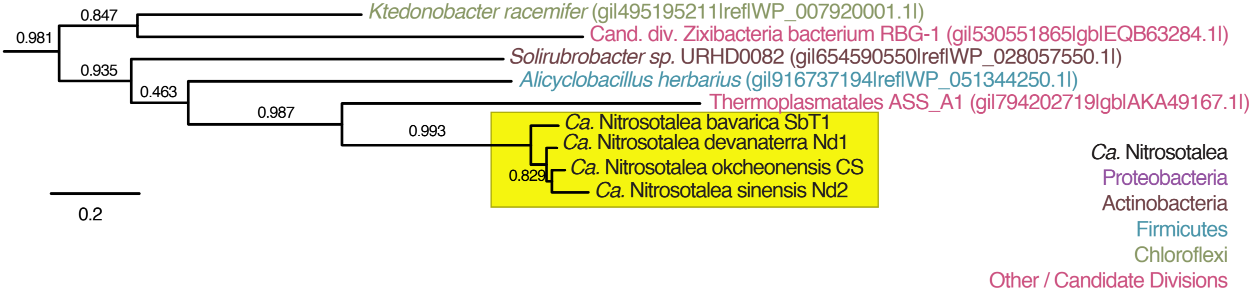

Figure S4. Maximum-likelihood phylogenetic tree of a putative divalent heavy-metal cations transporter (OG2531) that is *Ca.* Nitrosotalea-specific among Thaumarchaeota but also found distributed among other non-thaumarchaeotal lineages. Taxa are coloured according to phylum and accession numbers are provided. Genes from *Ca.* Nitrosotalea are highlighted. The complete sequence set was identified using *Ca.* Nitrosotalea amino acid sequences as queries for blastp searches against the NCBI nr database. Hits were screened for amino acid identity >30% over 70% of the length of any single *Ca.* Nitrosotalea query ortholog. The whole dataset consisted of four *Ca.* Nitrosotalea and 51 database hits. The five closest phylogenetic neighbours are shown and the outgroup consists of 46 additional database hits. The relationship of the ingroup with respect to individual outgroup clades remains unresolved. Proportional bootstrap support >0.5 is shown.

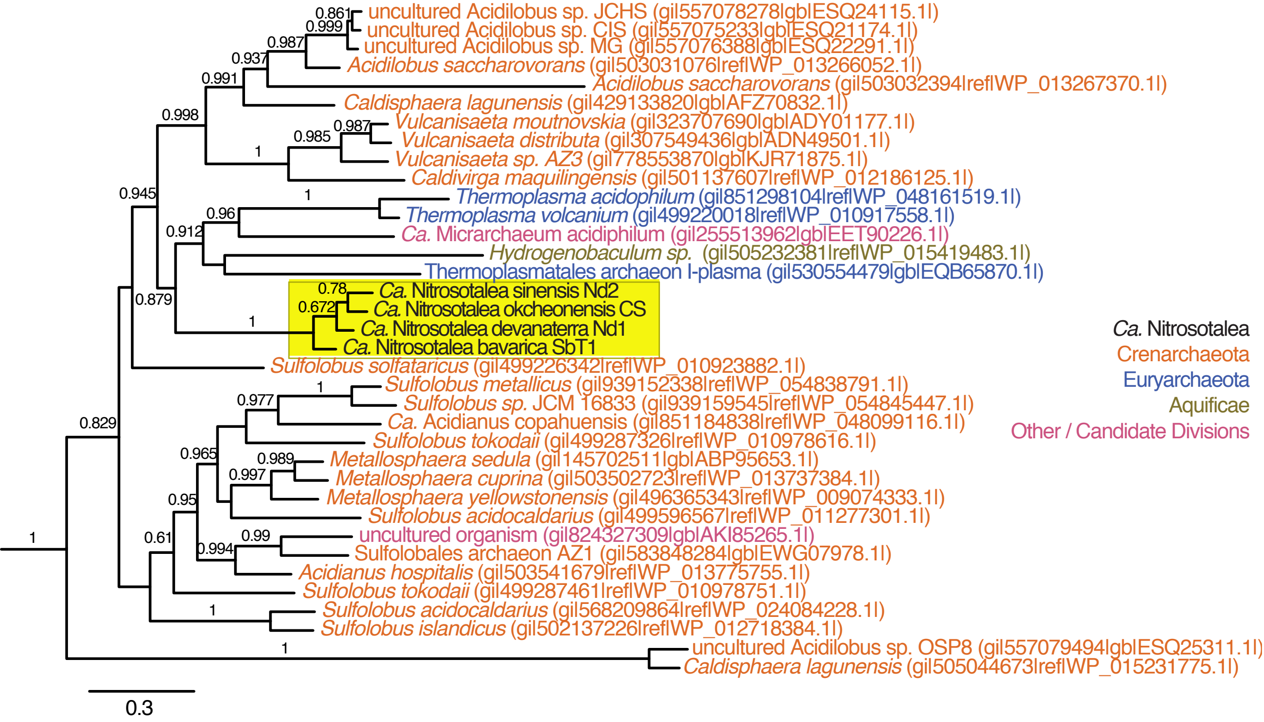

Figure S5. Maximum-likelihood phylogenetic tree of an Na+/solute symporter (OG2888) that is *Ca.* Nitrosotalea-specific among Thaumarchaeota but also found distributed among other non-thaumarchaeotal lineages. Taxa are coloured according to phylum and accession numbers are provided. Genes from *Ca.* Nitrosotalea are highlighted. The complete sequence set was identified using *Ca.* Nitrosotalea amino acid sequences as queries for blastp searches against the NCBI nr database. Hits were screened for amino acid identity >30% over 70% of the length of any single *Ca.* Nitrosotalea query ortholog. The whole dataset consisted of four *Ca.* Nitrosotalea and 462 database hits. The 32 closest phylogenetic neighbours are shown and the outgroup consists of 430 additional database hits. The relationship of the ingroup with respect to individual outgroup clades remains unresolved. Proportional bootstrap support >0.5 is shown.

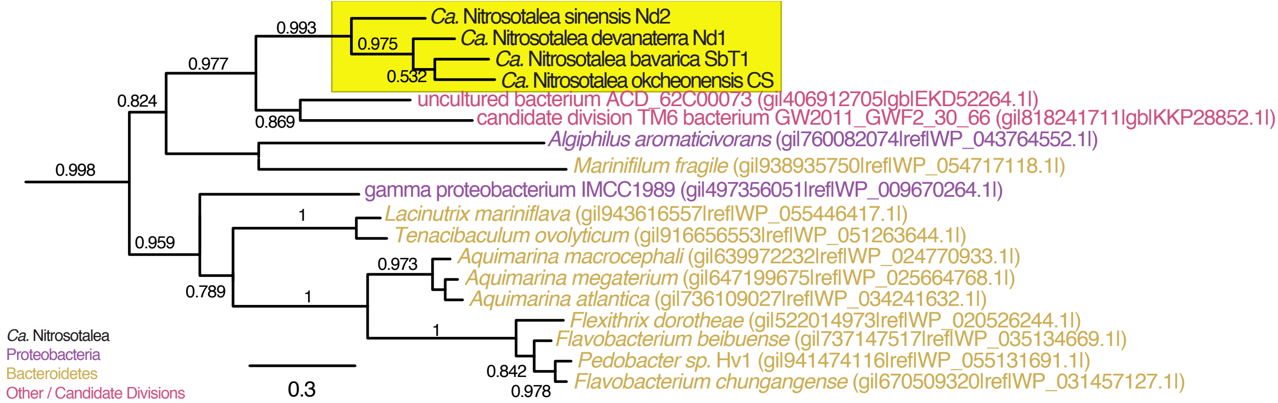

Figure S6. Maximum-likelihood phylogenetic tree of an FMN-dependent NADH-azoreductase (OG2912) that is *Ca.* Nitrosotalea-specific among Thaumarchaeota but also found distributed among other non-thaumarchaeotal lineages. Taxa are coloured according to phylum and accession numbers are provided. Genes from *Ca.* Nitrosotalea are highlighted. The complete sequence set was identified using *Ca.* Nitrosotalea amino acid sequences as queries for blastp searches against the NCBI nr database. Hits were screened for amino acid identity >30% over 70% of the length of any single Ca. Nitrosotalea query ortholog. The whole dataset consisted of four *Ca.* Nitrosotalea and 201 database hits. The 14 closest phylogenetic neighbours are shown and the outgroup consists of 183 additional database hits. The relationship of the ingroup with respect to individual outgroup clades remains unresolved. Proportional bootstrap support >0.5 is shown.

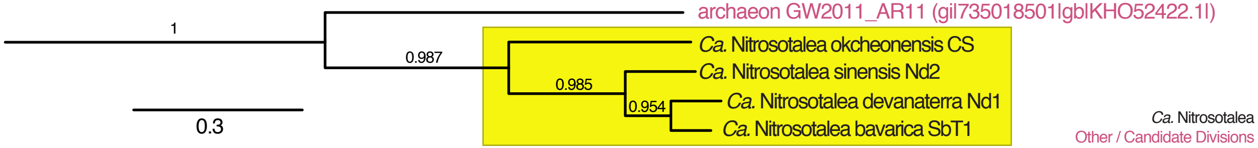

Figure S7. Maximum-likelihood phylogenetic tree of an NRAMP family Mn2+/Fe2+ transporter (OG2924) that is *Ca.* Nitrosotalea-specific among Thaumarchaeota but also found distributed among other non-thaumarchaeotal lineages. Taxa are coloured according to phylum and accession numbers are provided. Genes from *Ca.* Nitrosotalea are highlighted. The complete sequence set was identified using *Ca*. Nitrosotalea amino acid sequences as queries for blastp searches against the NCBI nr database. Hits were screened for amino acid identity >30% over 70% of the length of any single *Ca.* Nitrosotalea query ortholog. The whole dataset consisted of four *Ca.* Nitrosotalea and 477 database hits. The closest phylogenetic neighbour is shown and the outgroup consists of 476 additional database hits. The relationship of the ingroup with respect to individual outgroup clades remains unresolved. Proportional bootstrap support >0.5 is shown.

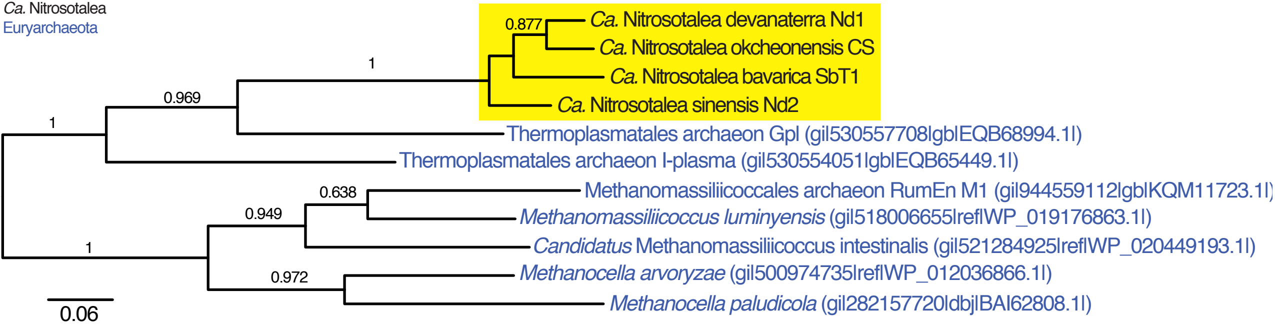

Figure S8. Maximum-likelihood phylogenetic tree of a putative phage protein (OG2932) that is *Ca.* Nitrosotalea-specific among Thaumarchaeota but also found distributed among other non-thaumarchaeotal lineages. Taxa are coloured according to phylum and accession numbers are provided. Genes from *Ca.* Nitrosotalea are highlighted. The complete sequence set was identified using *Ca.* Nitrosotalea amino acid sequences as queries for blastp searches against the NCBI nr database. Hits were screened for amino acid identity >30% over 70% of the length of any single *Ca.* Nitrosotalea query ortholog. The whole dataset consisted of four *Ca.* Nitrosotalea and 7 database hits. All eleven taxa are shown and the tree is midpoint-rooted. Proportional bootstrap support >0.5 is shown.

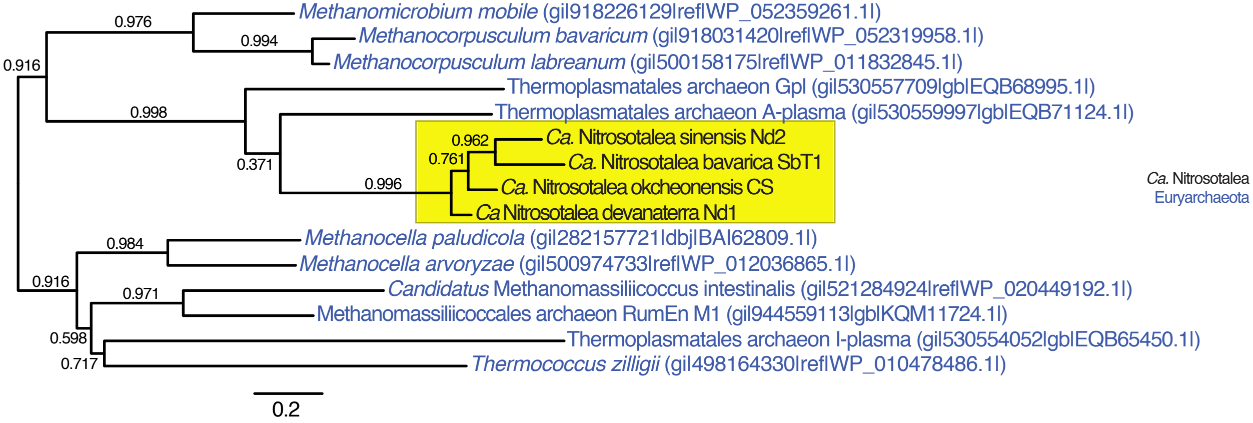

Figure S9. Maximum-likelihood phylogenetic tree of a coiled-coil motif protein of unknown function (OG2933) that is *Ca.* Nitrosotalea-specific among Thaumarchaeota but also found distributed among other non-thaumarchaeotal lineages. Taxa are coloured according to phylum and accession numbers are provided. Genes from Ca. Nitrosotalea are highlighted. The complete sequence set was identified using Ca. Nitrosotalea amino acid sequences as queries for blastp searches against the NCBI nr database. Hits were screened for amino acid identity>30% over 70% of the length of any single Ca. Nitrosotalea query ortholog.The whole dataset consisted of four Ca. Nitrosotalea and 11 database hits. All eleven taxa are shown and the tree is midpoint-rooted. Proportional bootstrap support >0.5 is shown.

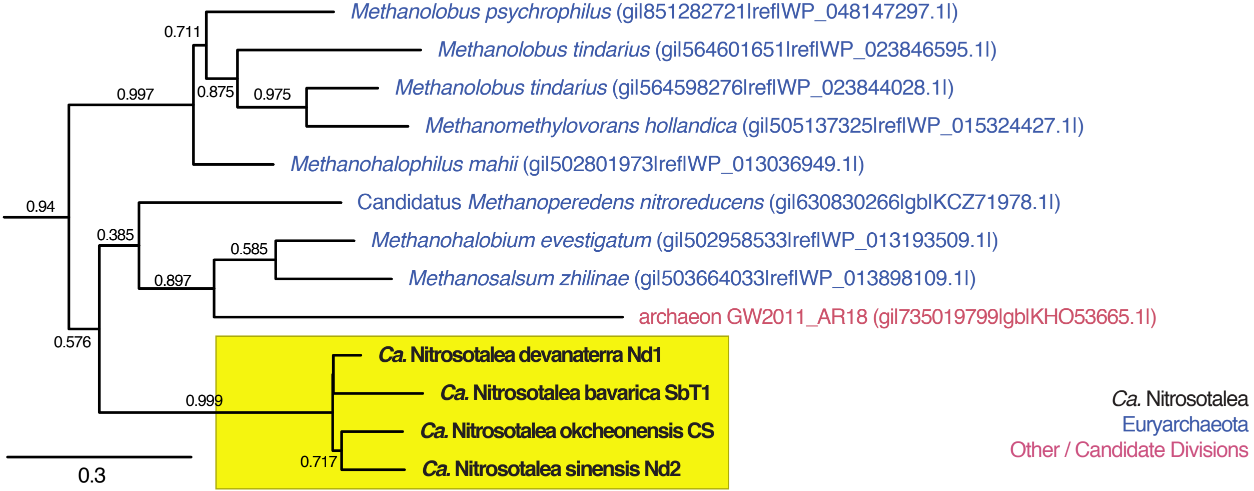

Figure S10. Maximum-likelihood phylogenetic tree of an FKBP-type peptidyl-prolyl cis-trans isomerase (OG2943) that is *Ca.* Nitrosotalea-specific among Thaumarchaeota but also found distributed among other non-thaumarchaeotal lineages. Taxa are coloured according to phylum and accession numbers are provided. Genes from *Ca.* Nitrosotalea are highlighted. The complete sequence set was identified using *Ca.* Nitrosotalea amino acid sequences as queries for blastp searches against the NCBI nr database. Hits were screened for amino acid identity>30% over 70% of the length of any single *Ca.* Nitrosotalea query ortholog. The whole dataset consisted of four *Ca.* Nitrosotalea and 513 database hits. The nine closest phylogenetic neighbour is shown here and the outgroup consists of 504 additional database hits. The relationship of the ingroup with respect to individual outgroup clades remains unresolved. Proportional bootstrap support >0.5 is shown.

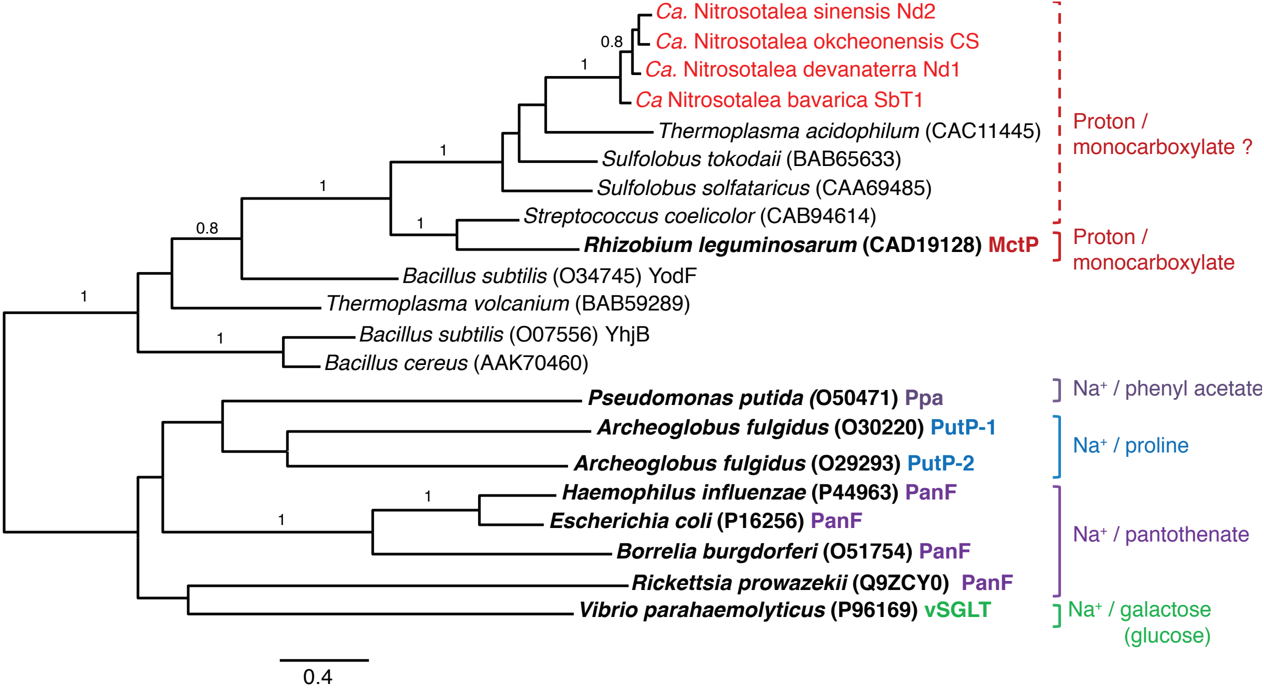

Figure S11. Maximum-likelihood phylogenetic tree of Na+/solute transporters based on sequences available in Jung (2002) plus the four *Ca.* Nitrosotalea sequences. Bootstrap values above 80% are indicated. Proteins from organisms shown in bold have been biochemically characterised. Accession numbers are provided in brackets.

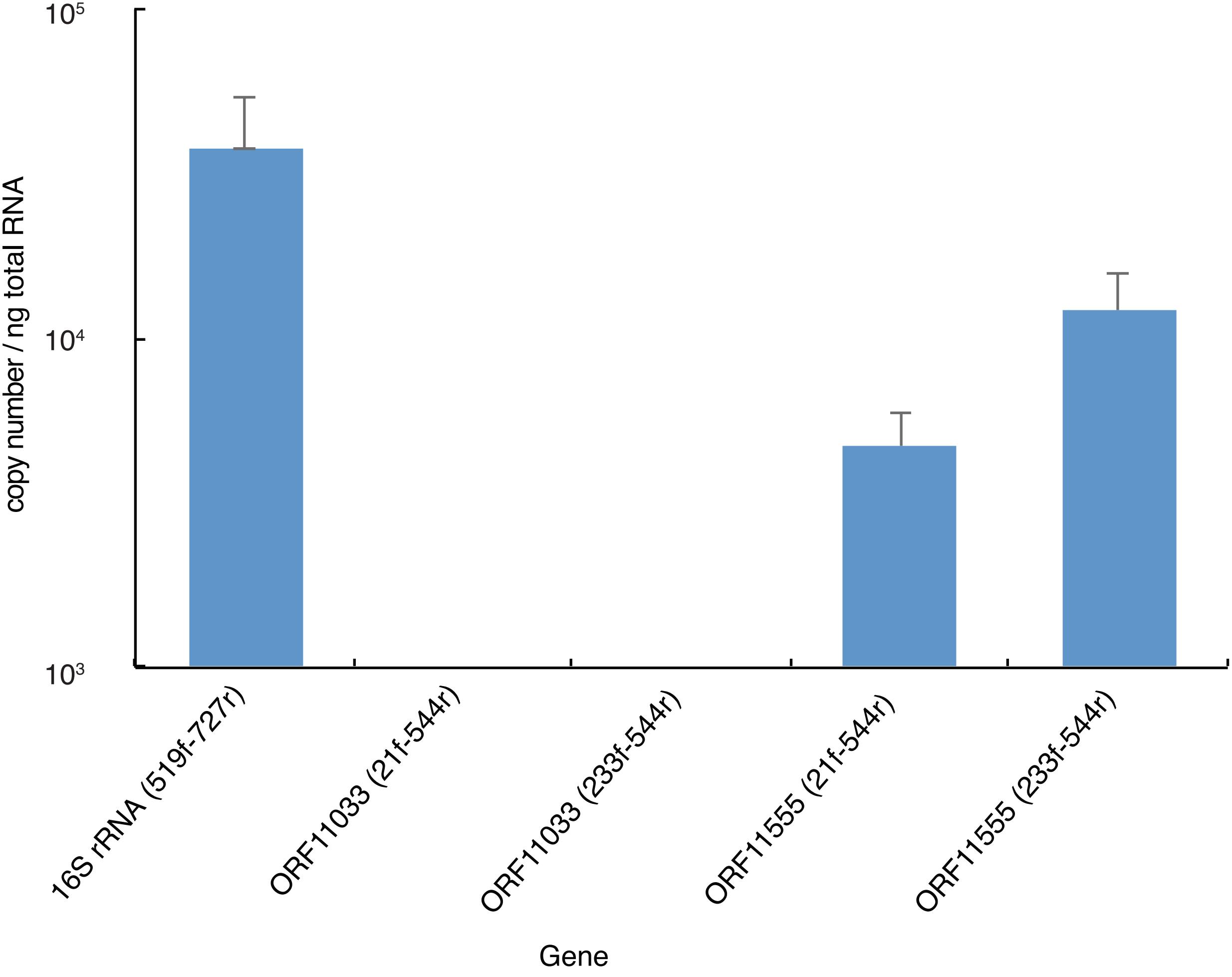

Figure S12. Transcript abundance of two *amoA* genes (ORF 11033 and 11555) and 16S rRNA in total RNA extracts from an exponentially growing culture of *Ca.* N. okcheonensis CS. Two different RT-qPCR assays were used for each *amoA* gene. Error bars are the standard deviation of three replicates.

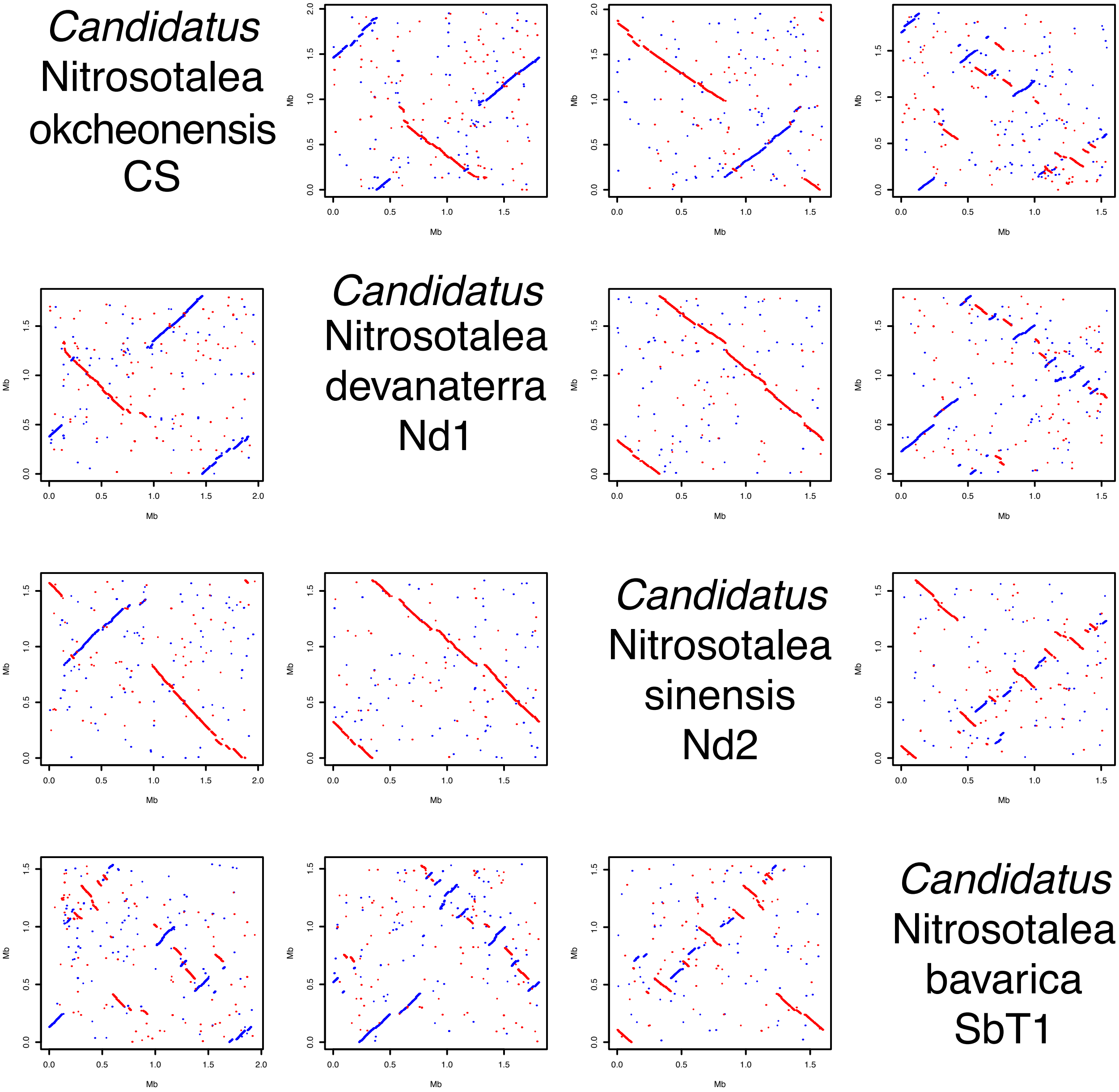

Figure S13. Mummer plots between *Ca.* Nitrosotalea genomes. Genomic coordinates are given in megabases (Mb). “Forward” alignments are shown in blue. Reverse-complement alignments are shown in red.

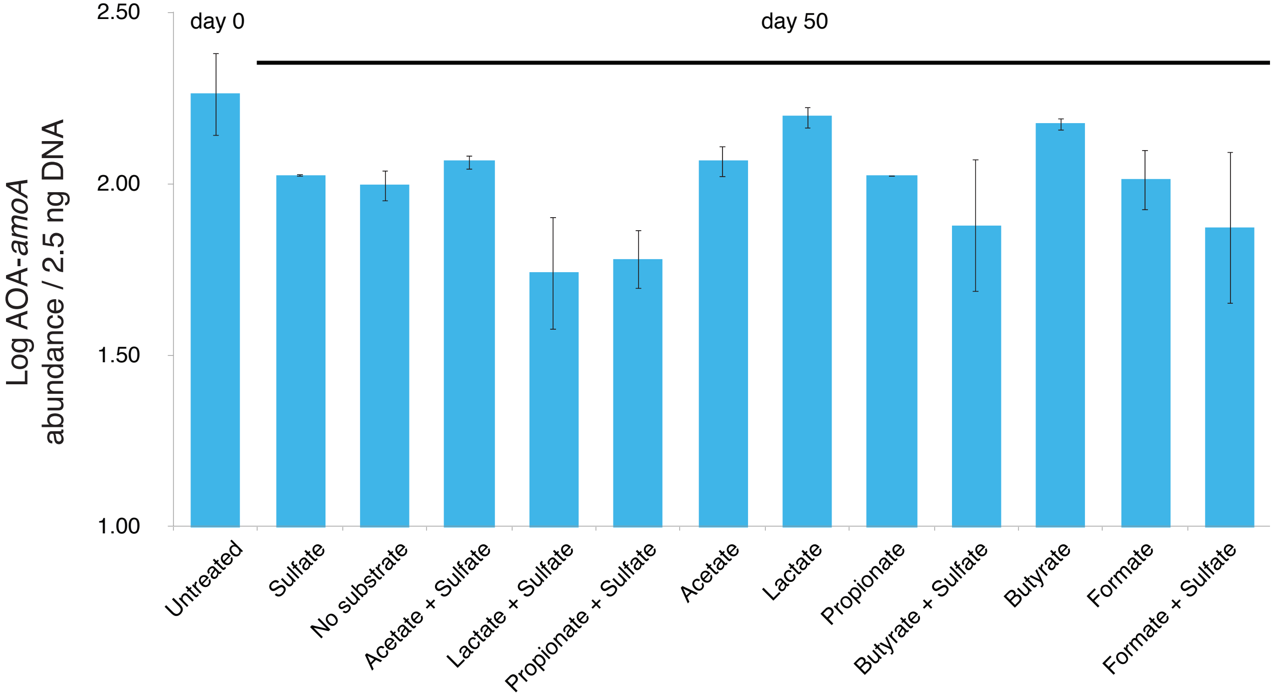

Figure S14. Abundance of thaumarchaeotal *amoA* genes in DNA extractions from anoxic Schlöppnerbrunnen peat soil microcosms incubated with different substrates (for details see Hausmann *et al*., 2016). No increase in, but persistence of thaumarchaeotal *amoA* genes was observed after 50 days of incubation in all treatments. Bar height corresponds to the mean and error bars are the standard deviation of three replicate measurements.

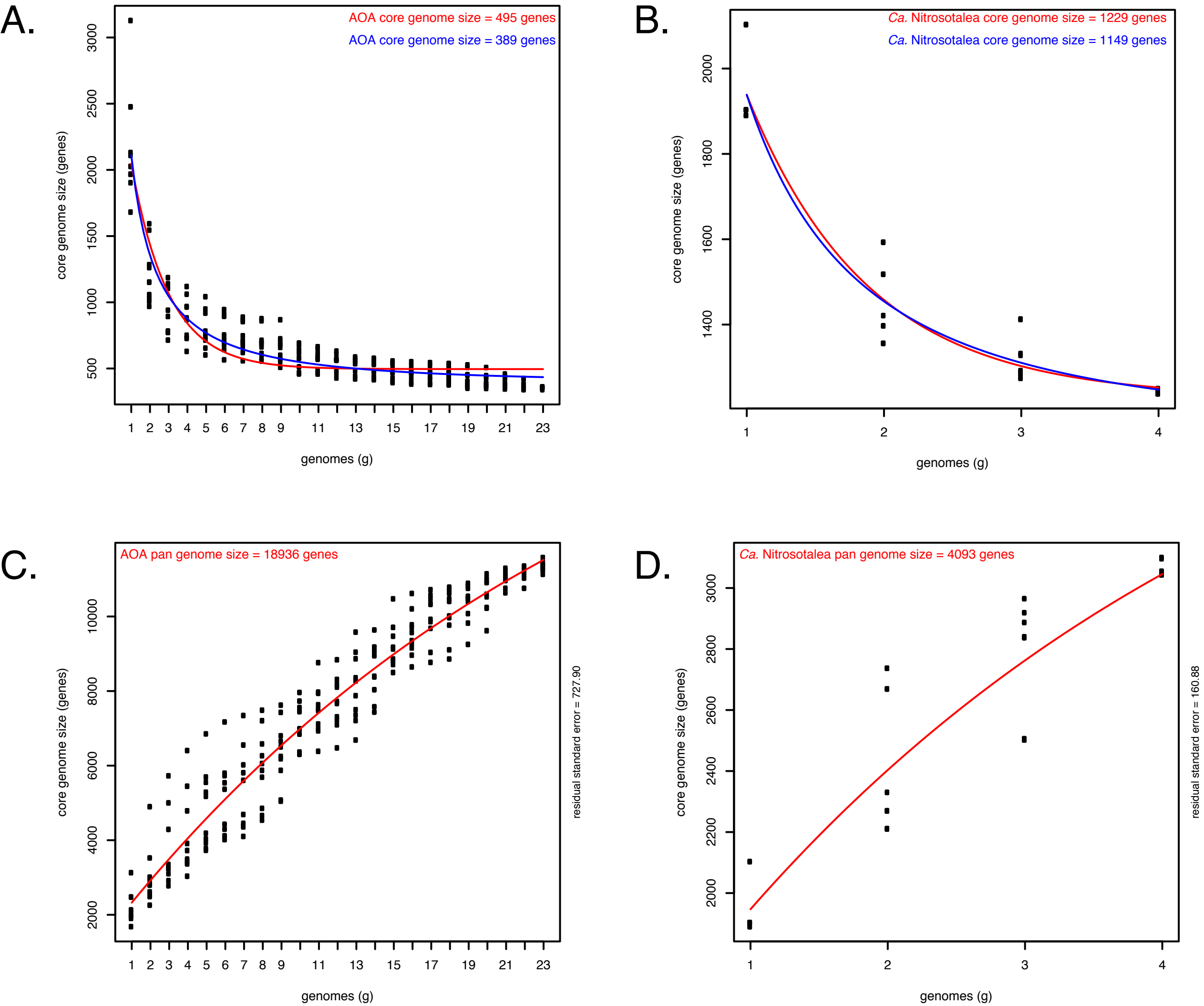

Figure S15. Theoretical core genome (A, B) and theoretical pangenome (C, D) sizes of 23 AOA (panel A, C) and four *Ca.* Nitrosotalea (B, D) strains. Random sampling was performed 10 times and the exponential models described in Tettelin et al. 2005 (red) and Willenbrock et al. 2007 (blue) were used to predict the size of core genomes (A and B) and pangenomes (C and D) extrapolated to infinite genomes sampled
